# PRG5 Knockout Precipitates Late-Onset Hypersusceptibility to Pilocarpine-Induced Juvenile Seizures by Exacerbating Hippocampal Zinc Signaling-Mediated Mitochondrial Damage

**DOI:** 10.3389/fnins.2021.715555

**Published:** 2021-08-27

**Authors:** Dandan Wang, Mei-fang Jin, Lili Li, Yueying Liu, Yuxiao Sun, Hong Ni

**Affiliations:** ^1^Division of Brain Science, Institute of Pediatric Research, Children’s Hospital of Soochow University, Suzhou, China; ^2^Department of Pediatrics, First Affiliated Hospital of University of Science and Technology of China, Hefei, China; ^3^Department of Pediatrics, North Branch, Affiliated Hospital of Jiangnan University, Wuxi, China

**Keywords:** zinc signaling, hippocampal mossy fiber sprouting, hypersusceptibility, PRG5, developmental seizure

## Abstract

**Introduction:**

Epileptogenesis is understood as the plastic process that produces a persistent reorganization of the brain’s neural network after a precipitating injury (recurrent neonatal seizures, for instance) with a latent period, finally leading to neuronal hyperexcitability. Plasticity-related genes (PRGs), also known as lipid phosphate phosphatase-related proteins (PLPPRs), are regulators of mitochondrial membrane integrity and energy metabolism. This study was undertaken to determine whether PRG5 gene knockout contributes to the delayed hypersensitivity induced by developmental seizures and the aberrant sprouting of hippocampal mossy fibers, and to determine whether it is achieved through the mitochondrial pathway. Here, we developed a “twist” seizure model by coupling pilocarpine-induced juvenile seizures with later exposure to penicillin to test the long-term effects of PRG5 knockout on seizure latency through comparison with wild-type (WT) mice. Hippocampal mossy fiber sprouting (MFS) was detected by Timm staining. In order to clarify the mechanism of the adverse reactions triggered by PRG5 knockout, hippocampal HT22 neuronal cultures were exposed to glutamate, with or without PRG5 interference. Mitochondrial function, oxidative stress indicators and zinc ion content were detected.

**Results:**

PRG5 gene knockout significantly reduced the seizure latency, and aggravated the lowered seizure threshold induced by developmental seizures. Besides, knockout of the PRG5 gene reduced the MFS scores to a certain extent. Furthermore, PRG5 gene silencing significantly increases the zinc ion content in hippocampal neurons, impairs neuronal activity and mitochondrial function, and exacerbates glutamate-induced oxidative stress damage.

**Conclusion:**

In summary, PRG5 KO is associated with significantly greater hypersusceptibility to juvenile seizures in PRG5^(–/–)^ mice compared with WT mice. These effects may be related to the hippocampal zinc signaling. The effects do not appear to be related to changes in MFS because KO mice with juvenile seizures had the shortest seizure latencies but exhibited less MFS than WT mice with juvenile seizures.

## Introduction

Approximately 70 million people worldwide suffer from epilepsy, half of which are children, in whom the prevalence is approximately 0.5–0.8% (Málaga et al., 2019). Although many third-generation anti-seizure drugs (ASDs) (such as lacosamide, esikazepine acetate, brivaracetam, perampanel, retigabine, everolimus, and cannabidiol) have been included in clinical practice, only approximately 50% of newly diagnosed patients respond to the first anti-seizure drug prescribed, and approximately 30% of epilepsy patients continue to have seizures and have anti-seizure drug-refractory epilepsy (Málaga et al., 2019; Bresnahan et al., 2020). In pediatric neurology, many children who suffer from neonatal seizures develop epilepsy in adulthood. However, anticonvulsants aggravate brain damage, such as white matter damage (Kaushal et al., 2016). In recent years, significant progress has been made in understanding the effects of non-anticonvulsant drug treatment on epilepsy, particularly a ketogenic diet (KD) and autophagy modulators. The hippocampal phospholipase signaling pathway, especially the PRG family (plasticity-related genes, PRGs), may be a target for the protection of KD or autophagy inhibitors against long-term brain damage after developmental seizures (Ling et al., 2019; Zheng et al., 2020).

PRGs is a subclass of the lipid phosphate phosphatase (LPP-) superfamily (PRGs/LRPs, PRG1-5 or LPA1-5, Plppr1-5), which mediates lipid phosphate phosphatase activity and neuronal plasticity (Brauer and Nitsch, 2008; Trimbuch et al., 2009). PRG1 regulates bioactive lipids of postsynaptic density, is highly expressed at postsynaptic density during development and regeneration, and modulates the level of synaptic lysophosphatidic acid (LPA) (Brauer and Nitsch, 2008; Petzold et al., 2016). Notably, Trimbuch et al. (2009) described seizure generation in mice that lack PRG1. They found that deletion of PRG-1 in mice led to epileptic seizures and augmentation of EPSCs, suggesting an important role of PRG-1 in the modulatory control of hippocampal excitability related to epilepsy and brain damage. Our previous study also found that PRG-1 and ZnT-3 mRNA protein levels in the hippocampus were upregulated at 14 days after flurothyl-induced neonatal seizures, which is in parallel with seizure-induced hippocampal mossy fiber sprouting (MFS) and cognitive deficits (Ni et al., 2009, 2010). Subsequently, we examined the effects of E-64d (an autophagy inhibitor) on hippocampal aberrant MFS following penicillin-induced recurrent epilepticus. The results showed that, compared with the seizure group (EXP1) not treated with E64d, not only PRG1 but also PRG5 mRNA levels in the hippocampus of seizure rats pretreated with E-64d (EXP2) were significantly downregulated, which was in parallel with reduced MFS in the hippocampus (Ni et al., 2013). The cloning of PRG5 (also known as Plppr5) was reported by Broggini et al. (2010). PRG5 is only distributed in the nervous system. PRG5 is distributed in the plasma membrane of neurons and is specifically involved in filopodia and axon growth, as well as dendritic spine formation. siRNA-mediated knockdown of PRG5 hinders the growth of axons and interferes with the formation of filopodia (Broggini et al., 2010). However, the role of PRG5 in epileptogenesis, especially developmental-induced mossy fiber spouting and seizure susceptibility, has not been extensively studied.

Epileptogenesis is the plastic process that refers to the development and extension of tissue capable of generating spontaneous seizures, resulting in the development of an epileptic condition and/or progression of epilepsy after the condition is established. During epileptogenesis, the hippocampus, as the initiation area of many epilepsy patients, undergoes plasticity changes, which are mainly manifested in mossy fiber sprouting (MFS) and alterations in dendritic branching, spine density and shape (Sierra et al., 2015). Hippocampal MFS induced by developmental seizures is a typical phenomenon of aberrant axon growth in response to seizures, which could be detected by using a zinc-detecting histologic technique (Timm). Interestingly, MFS is an active phenomenon that is reversible. Mitophagy-mediated zinc homeostasis via mitochondrial activation may be a potential mechanism (Ni et al., 2016, 2019; Li et al., 2018). In addition, our previous studies have shown that developmental seizures cause long-term abnormal expression of hippocampal zinc transporters and plasticity-related genes (PRG1 and PRG-3), and the high correlation between these molecules is also destroyed (Ni et al., 2009, 2010; Tian et al., 2016), indicating that both PRG and zinc signaling are involved in the long-term hippocampal metabolic abnormalities after neonatal seizures. However, the effect of PRG5 on seizure-induced brain damage and its relationship with zinc ion signals remain unclear. Therefore, we also further revealed the internal connection between PRG5, mitochondrial damage and zinc ion signal through *in vitro* cell experiments. Here we report for the first time the segregation phenotype of PRG5^–/–^ mice with seizure latency and aberrant sprouting of hippocampal mossy fibers. We performed a “twist” seizure model to determine the seizure latency in PRG5^–/–^ mice. Our results suggest a role for PRG5 and zinc signaling in the underlying mechanisms of epileptogenesis.

## Materials and Methods

### Animal Preparation

The generation of PRG5 knockout mice was conducted at the Nanjing Biomedical Research Institute of Nanjing University (Nanjing, China). The gRNA was designed and transcribed *in vitro* using CRISPR/Cas9 technology. Then, Cas9 and gRNA were injected into fertilized mouse eggs at the same time (see Table 1 for the sequence information for gRNA). The Cas9 protein binds to the target site under the guidance of gRNA, causing DNA double-strand breaks, resulting in the deletion of the base sequence of the target site and ultimately achieving systemic gene knockout (Sun et al., 2021). The strain backgrounds of PRG5 knockout mice and WT wild-type mice are both C57BL/6JNju (see Table 2 for strain information of knockout mice).

**TABLE 1:** Sequence information of gRNA.

**gRNA name**	**gRNA sequence**	**PAM**
gRNAl	5*′*-ACCACTAGGCAG TAGAGACT-3*′*	GGG
gRNA2	5*′*-TGTGAGGACAATTGGCTCTA-3***′***	AGG
gRNA3	*5*′-GGG CTTTGTCGTGGGTG G C G-3***′***	GGG
gRNA4	*5*′-CACAGTCTCGTGGGAGGGCG-3***′***	GGG

**TABLE 2:** Strain information of knockout mice.

**Name**	**Code**
Strain number	T004790
Source of strain	GemPharmatech Co., Ltd.
Strain name	B6/JNju-Plppr5em1Cd/Nju u
Strain background	C57BL/6JNJu
Strain type	Cas9-KO

We bred male heterozygous PRG5 knockout mice (PRG5±) and female heterozygous PRG5 knockout mice (PRG5±) in a 1:2 breeding cage. Both PRG5 knockout mice and littermate control wild-type mice were derived from the progeny of heterozygous PRG5 knockout mice. To identify whether the PRG5 protein is knocked out in KO mice, we first cut 3–5 mm tail tip tissue from the offspring mice born at the age of 3 days, and used PCR to perform genotype identification, and compare the size of the DNA products of the mice to be identified: Wild-type mice (PRG5^–/–^) = 439 bp, homozygous PRG5 knockout mice (PRG5^–/–^) = 414 bp, heterozygous PRG5 knockout mice (PRG5 ±) = 414 bp + 439 bp. (The process was completed by the Nanjing Institute of Biomedicine, Nanjing University) (Sun et al., 2021).

All procedures were reviewed and approved by the Institutional Animal Care and Use Committee of Soochow University. We took adequate measures to minimize animal suffering, and the sample size was based on the sample size used in our group’s previous publications and other similar studies (Companys-Alemany et al., 2020).

### Group Allocation

Thirty 25-day-old (P25) wild-type mice were randomly divided into two groups: WT group (*n* = 15) and WT + SE group (*n* = 15); another 30 25-day-old (P25) PRG5 knockout mice were randomly divided into 2 groups: KO group (*n* = 15) and KO + SE group (*n* = 15). (KO represents PRG5^–/–^ mice without seizure injury, WT means wild-type mice without seizure injury, KO + SE represents PRG5^–/–^ mice with seizure injury, WT + SE means wild-type mice with seizure injury).

**FIGURE 1:**
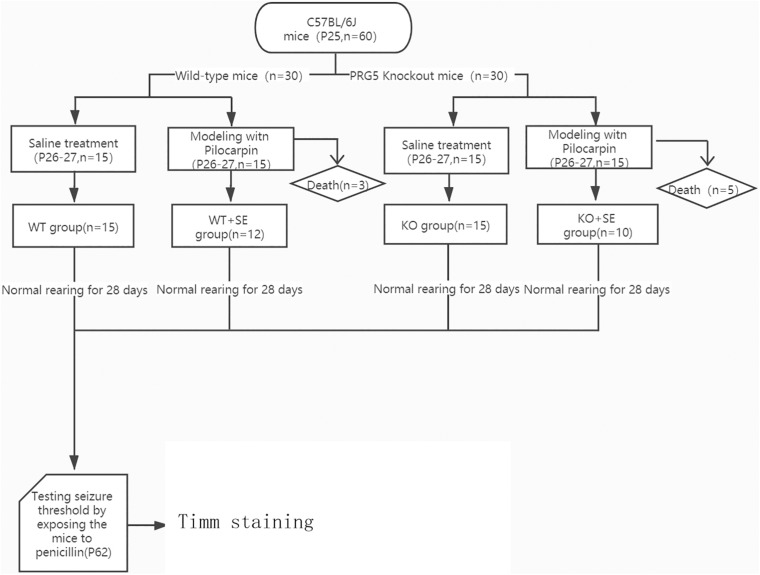
Schematic diagram of the experimental process.

A schematic diagram of the entire experiment is shown in Figure 1.

### Animal Model of Juvenile Seizure

The induction of juvenile seizures was performed as described previously (Ni et al., 2018). Each mouse in the WT + SE group and KO + SE group was injected with lithium chloride (127 mg/kg, i.p.) at P26. Then, after 24 h (P27), animals were injected with pilocarpine (320 mg/kg, i.p.) to induce juvenile seizure. Scopolamine methyl chloride hydrobromide was injected (1 mg/kg, i.p.) 30 min before the injection of pilocarpine to antagonize the peripheral effect of pilocarpine. The control mice in the WT group and KO group received saline injections at the same time and the same handling as the seizure groups. After the injection, the seizures were observed according to the Racine classification (Racine, 1972). Epileptic seizures reaching grade IV and above were considered successful induction, and the mice that did not reach grade IV were eliminated. All animals in the WT + SE group and KO + SE group reached Racine stage 4 or higher during the modeling process. After successful modeling, 12 survived in the WT + SE group (*n* = 12), and 10 survived in the KO + SE group (*n* = 10).

### Seizure Latency

Seizure susceptibility was studied by exposing the mice to penicillin using a procedure modified from one previously described procedure (Ni et al., 2018). All mice from each group were injected with penicillin (5.1 × 10^6^ U/kg/d, i.p.) on postnatal day 62. The time to the first seizure after penicillin injection was recorded, which was the seizure latency (min) (seizure latency). The observation time was 2 h.

Mice were anesthetized, and brains were removed for Timm staining/Western blotting as soon as the animals reached Racine Stage 4. Five mice in each group were randomly selected for Western blot detection, and five were used for Timm’s staining.

### Timm Staining

At the end of the seizure latency test on P62, five mice from each group were randomly selected and given i.p. injection of chloral hydrate at a dose of 1 ml/100 g. After the anesthesia was complete, each group was perfused through the heart with 0.9% saline followed in order by PBS solution, precooled Na2S-PBS solution, precooled 4% paraformaldehyde solution, and precooled Na2S-PBS solution. The method used for Timm staining and semiquantitative scoring of the hippocampal CA3 area and dentate gyrus (DG) MFS has been described previously (Ni et al., 2009).

The person scoring the Timm staining was blind to the treatment group. The scoring criteria in the supragranular region of dentate gyrus or in the stratum pyramidal or stratum oriens of CA3 are divided into 6 levels from 0 to 5, 0: no granules; 1: occasional granules; 2: occasional to moderate granules; 3: prominent granules; 4: prominent granules in the stratum pyramidal or stratum oriens occurring in near-continuous distribution along the entire CA3 region, or highly concentrated band of granules appearing either in continuous or near-continuous distribution; 5: continuous or near continuous dense laminar band of granules in the stratum pyramidal or stratum oriens along the entire CA3 region, or continuous dense laminar band of granules from crest to tip of dentate (Sogawa et al., 2001).

### Cell Lines

The HT22 mouse hippocampal neuron cell line was obtained from the Cell Bank of the Institute of Cell Biology, Chinese Academy of Sciences.

### Cell Culture Conditions

HT22 cells were cultured in DMEM supplemented with 10% fetal bovine serum, 100 U/ml penicillin, and 100 mg/ml streptomycin in humidified air at 37°C with 5% CO_2_.

### Drug Treatment and Grouping

The shControl group, shControl + Glutamate group, shPRG5 group and shPRG5 + Glutamate group were established. Each group is processed as follows:

(a) shControl group: After 72 h of infecting cells with no-load lentivirus, change to normal medium. shControl source/sequence: 5′-TTCTCCGAACGTGTCACGT-3′.
(b) shControl + Glutamate group: 72 h after the cells are infected with no-load lentivirus, replaced with a culture medium containing 5 mM (Song et al., 2017) glutamic acid (with serum-containing medium configuration) and cultured for 24 h.
(c) shPRG5 group: 72 h after shPRG5 lentivirus infects the cells, change to normal medium. shPRG5 source/sequence: 5′-GCAATTAGCCACAAGAGAT-3′.
(d) shPRG5 + Glutamate group: 72 h after shPRG5 lentivirus infects the cells, replaced with 5 mM glutamate (serum-containing medium configuration) culture medium and cultured for 24 h.

### Analysis of Mitochondrial Membrane Potentials Using JC-1

After the experimental grouping and treatment, the cells were collected by trypsin digestion, resuspended in 0.5 ml cell culture medium and added the mitochondrial membrane potential sensitive probe JC-1 (Beyotime, Shanghai, China) staining working solution 0.5 ml, shaking and mixing. Cells were incubated in an incubator for 30 min at a final concentration of 2 μM in PBS and washed twice with PBS. JC-1 aggregate was measured at the FL-2 channel, and green fluorescence (JC-1 monomer) was measured at the FL-1 channel. The data were analyzed using the FlowJo analysis software, and the results are displayed in a dot plot of J-aggregate red fluorescence (y-axis) against JC-1 green fluorescence (x-axis) (Ma et al., 2017).

### Measurement of Intracellular Zinc Ions Concentration

The cells were seeded in a small confocal dish and cultured overnight. After washing the cells twice with HBSS in each group, 250 μl of 12 μM Zinquin ethyl ester solution (diluted in HBSS, Dojindo Molecular Technologies, Kumamoto, Japan) was added to the cells and incubated at 37°C for 1 h. After washing the cells twice with HBSS, a laser confocal microscope was used to detect the fluorescence intensity of the cells (Jin et al., 2018).

### Statistical Analysis

The data were analyzed with two-way ANOVAs with *post hoc* Bonferroni’s multiple comparisons tests using SPSS 23.0 software. Data are presented as the mean ± SD. Statistical significance was considered as *P* < 0.05.

## Results

### Seizure Latency

**FIGURE 2:**
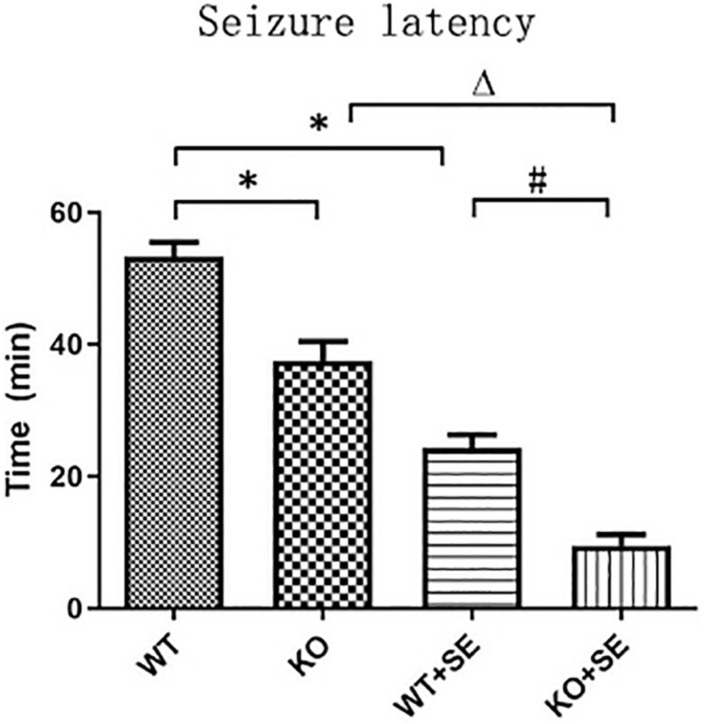
PRG5 KO increased seizure susceptibility, as assessed in the seizure latency test, in which the seizure latencies of the two knockout groups were significantly lower than those of the corresponding control group (KO vs. WT, KO + SE vs. WT + SE). ^∗^*P* < 0.05, compared to the WT group, ^△^*P* < 0.05 compared to the KO group, ^#^*P* < 0.05 compared to the WT + SE group.

The reduction in seizure latency represents an increase in brain excitability. At P62, all mice had seizures after penicillin injection. The seizure latencies of the two pilocarpine-treated groups were significantly lower than those of the corresponding control group (WT + SE vs. WT, KO + SE vs. KO) [two-way ANOVA; Genotype × Seizure *F*(1, 48) = 0.047, *P* = 0.829; Genotype *F*(1, 48) = 40.056, *P* < 0.001; Seizure *F*(1, 48) = 140.012, *P* < 0.001; *post hoc* Bonferroni’s multiple comparisons test, *P* < 0.05; WT group: *n* = 15, KO group: *n* = 15, WT + SE group: *n* = 12, KO + SE group: *n* = 10]. This is consistent with previous studies, indicating that early developmental seizures increase brain excitability and susceptibility to epilepsy. Interestingly, the seizure latencies of the two knockout groups were significantly lower than those of the corresponding control group (KO vs. WT, KO + SE vs. WT + SE), suggesting that PRG5 knockout changes the balance of excitability and inhibitory activity in the brain by affecting plasticity signaling, making animals more prone to seizures (Figure 2).

### Timm Staining

**FIGURE 3:**
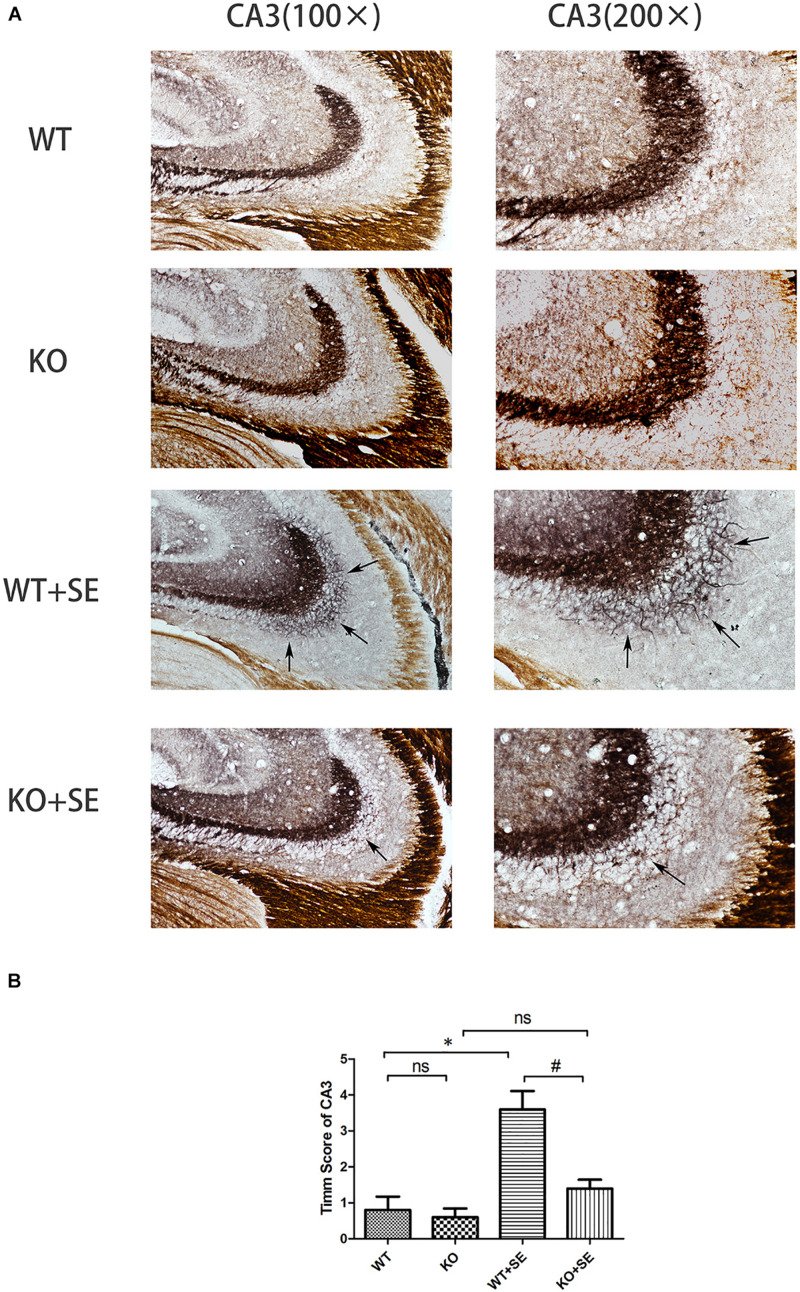
Juvenile seizures increased mossy fiber sprouting (indicated by the arrows) in the pyramidal cell layer of the hippocampal CA3 area of WT mice, but this effect was attenuated in KO mice. **(A)** Timm staining in CA3 subfield; **(B)** Timm score analysis. ^∗^*P* < 0.05, compared to WT group, ^#^*P* < 0.05 compared to WT + SE group, ns means no statistical significance.

**FIGURE 4:**
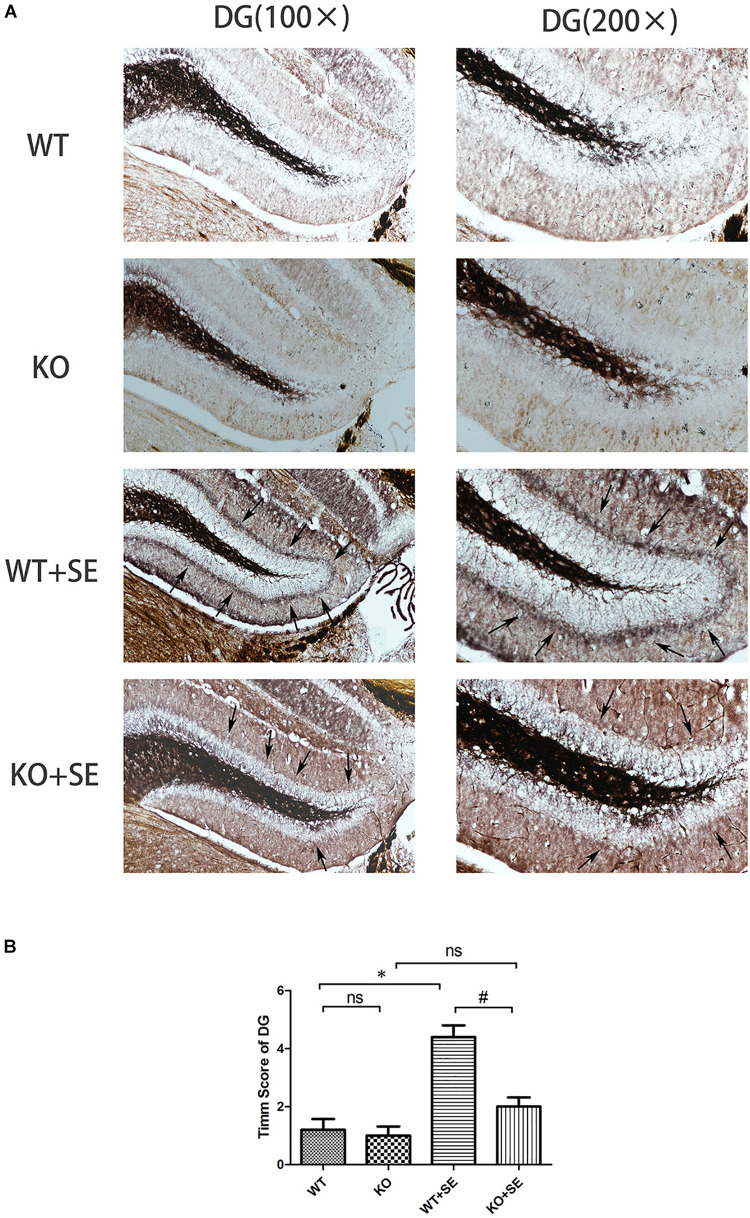
Juvenile seizures increased mossy fiber sprouting (indicated by the arrows) in the inner molecular layer of the dentate gyrus of WT mice, but this effect was attenuated in KO mice. **(A)** Timm staining in dentate gyrus subfield; **(B)** Timm score analysis. ^∗^*P* < 0.05, compared to WT group, ^#^*P* < 0.05 compared to WT + SE group, ns means no statistical significance.

Timm staining showed that the mossy fibers in the WT + SE group grew significantly toward the pyramidal cell layer of the hippocampal CA3 area and the inner molecular layer of the granular cells of the dentate gyrus. The sprouting scoring analysis further showed that the scores of CA3 and DG areas of WT + SE group were significantly higher than those of the WT group [two-way ANOVA; CA3: Genotype × Seizure *F*(1, 16) = 7.692, *P* = 0.014; Genotype *F*(1, 16) = 11.077, *P* = 0.004; Seizure *F*(1, 16) = 24.923, *P* < 0.001. DG: Genotype × Seizure *F*(1, 16) = 9.68, *P* = 0.007; Genotype *F*(1, 16) = 13.52, *P* = 0.002; Seizure *F*(1, 16) = 35.28, *P* < 0.001, *n* = 5/each group]. PRG5 gene knockout significantly reduced the MFS score, which was specifically reflected in the fact that PRG5 gene knockout significantly reduced the postconvulsive Timm score of the KO + SE group compared with the WT + SE group [*post hoc* Bonferroni’s multiple comparisons test, *P* < 0.05.]. In addition, although the MFS score of the KO group was not significantly different from that of the WT group, the MFS scores of the CA3 and DG areas of the KO group were 25 and 16.7% lower than those of the WT group, respectively (Figures 3, 4).

### Effect of PRG5 Gene Silencing on Glutamate-Induced Mitochondrial Membrane Potential Changes Using JC-1

The increase of mitochondrial membrane potential will cause JC-1 to accumulate in the mitochondrial matrix and produce red fluorescence. When the mitochondrial membrane potential is low, JC-1 cannot accumulate in the mitochondrial matrix, resulting in green fluorescence. The change of JC-1 from red fluorescence to green fluorescence is an indicator of early cell apoptosis.

**FIGURE 5:**
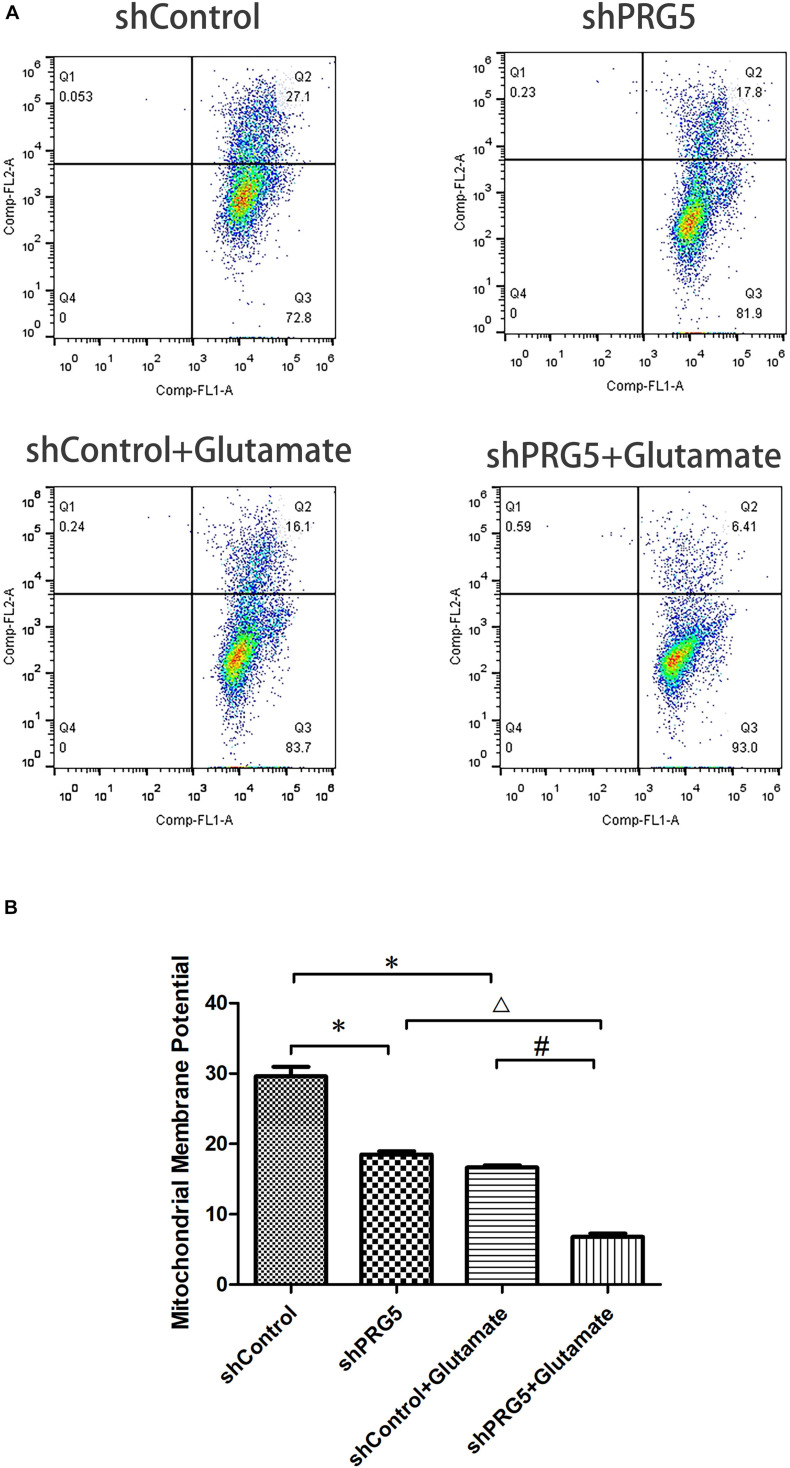
The effect of PRG5 gene silencing and glutamate treatment on the changes of mitochondrial membrane potential by JC-1. **(A)** Flow cytometric diagram of cell mitochondrial membrane potential in each group. **(B)** Analysis of cell mitochondrial membrane potential in each group. ^∗^*P* < 0.05 compared with shControl group, ^△^ *P* < 0.05 compared with shPRG5 group, ^#^*P* < 0.05 compared with Control + Glutamate group (*n* = 3).

The results of flow cytometry showed that both plppr5 gene silencing and glutamate treatment significantly reduced the mitochondrial membrane potential when compared with the shControl group (*P* < 0.05). Moreover, the mitochondrial membrane potential of the shPRG5 + Glutamate group was significantly lower than that of the shControl + Glutamate (*P* < 0.05) and the shPRG5 + Glutamate group, indicating that PRG5 gene silencing can reduce the mitochondrial membrane potential of HT22 cells treated with glutamate (Figure 5).

### Measurement of Intracellular Zinc Ions Concentration

**FIGURE 6:**
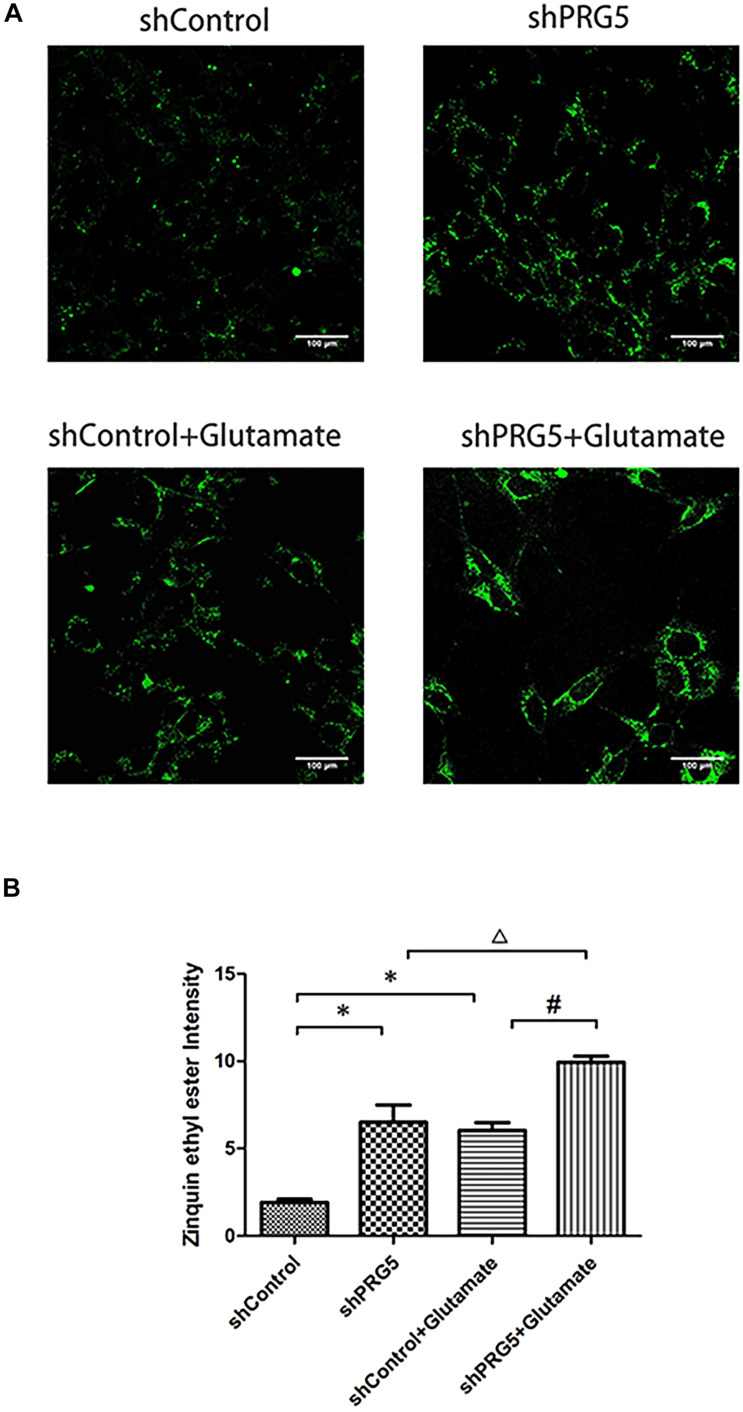
The effect of PRG5 gene silencing and glutamate treatment on the cellular zinc ion levels in HT22 cells. **(A)** The fluorescence intensity of Zinquin ethyl ester in HT22 cells; **(B)** the quantitative analysis of the average fluorescence intensity of Zinquin ethyl ester in HT22 cells. ^∗^*P* < 0.05 compared with shControl group, ^△^ *P* < 0.05 compared with shPlppr5 group, ^#^*P* < 0.05 compared with Control + Glutamate group (*n* = 3).

PRG5 gene silencing and glutamic acid treatment both significantly increased the cellular zinc ion level. As shown in Figure 6, the zinc ion content in the shControl + Glutamate and shPRG5 + Glutamate groups was markedly higher than that in the corresponding shControl and shPRG5 groups, respectively (*P* < 0.05). Moreover, the zinc ion content was significantly elevated in the shPRG5 group compared to the shControl group (*P* < 0.05). Furthermore, the zinc ion content was significantly higher in the shPRG5 + Glutamate group than in the shControl + Glutamate (*P* < 0.05).

## Discussion

Here, our *in vivo* results describe the outcomes of wild-type (WT) and PRG5 KO mice in young adulthood following pilocarpine-induced juvenile seizures, focusing on seizure threshold and hippocampal MFS. The main findings were that PRG5 KO mice, relative to WT controls, had shorter seizure latency times following penicillin administration. Regardless of genotype, juvenile seizures reduced seizure latency. Interestingly, seizures increased MFS in the WT mice, but this effect was attenuated in the KO mice.

Of note, regardless of juvenile seizures, PRG5 knockout resulted in reduced seizure latency. Specifically, the seizure latency of the two gene knockout groups was significantly lower than that of the corresponding control group (KO vs. WT, KO + SE vs. WT + SE). Since shortened seizure latency indicates increased brain excitability, this result suggests that PRG5 gene knockout may change the balance of brain excitability and inhibitory activity by regulating plasticity-related signals, thereby making animals more prone to seizures. The key mechanism of PRG5’s role in regulating brain excitability and seizure latency may be bioactive lipid phosphate. As shown by Trimbuch et al. (2009), PRG-1 regulates synaptic excitatory transmission through lipid phosphate-mediated signal transduction. There was previous report of aggravation of chronic stress effects on hippocampal neurogenesis and spatial memory in lysophosphatidic acid (LPA) receptor knockout mice (Castilla-Ortega et al., 2011). LPA also induced a decrease in mitochondrial membrane potential. Pretreatment of neurons with cyclosporin A protected against the LPA-induced decrease in mitochondrial membrane potential and neuronal apoptosis (Holtsberg et al., 1998). Moreover, we recently reported that cyclosporin A significantly reduced intracellular zinc ion content and corrected glutamate-induce cytotoxicity in mouse HT22 hippocampal neurons (Jin et al., 2018; Wang et al., 2019). Therefore, the bioactive lipid phosphates might play a key role in seizure latency in the PRG5 KO mouse brain after juvenile seizures, which merit further investigation by using PRG-5/LPA receptor-deficient animals.

The PRG family is a set of five brain-specific integral membrane proteins that are part of the lipid phosphate phosphatase superfamily. We previously examined the effect of E-64d (a calpain and autophagy inhibitor) on hippocampal aberrant MFS in a developmental rat model of penicillin-induced recurrent epilepticus. An interesting finding is that while E-64d obviously suppressed the aberrant MFS in the supragranular region of dentate gyrus and CA3 subfield of hippocampus, E-64d-pretreated seizure rats (EXP2) showed a significant downregulation of mRNA expression of PRG-1, PRG-3, and PRG-5 in hippocampus when compared with non-E64d-treated seizure rats. These results suggest that PRGs and sprouting may have some internal connection (Ni et al., 2013). PRG5, specifically, is involved in filopodia and axon growth, as well as dendritic spine formation (Broggini et al., 2010). However, there is no report on the direct effect of PRG5 on sprouting. Here, we investigated for the first time the role of PRG5 in aberrant sprouting of hippocampal mossy fibers in PRG5^–/–^ mice. We found that PRG5 knockout significantly reduced the postconvulsive MFS Timm staining score of wild-type mice (comparison between KO + SE group and WT + SE group); the MFS score of the non-seizure KO group with PRG5 knockout (KO) also decreased to a certain extent (compared with the WT group). This is consistent with the study of Broggini et al. (2010) They found that PRG5 promoted the formation of neurites and filopodia of primary neurons and that PRG5 silencing weakened the formation and growth of neurites (Broggini et al., 2010). These results highlight the possibility that PRG5 plays a role in promoting hippocampal mossy fiber axon regeneration. It should be mentioned that repeated sensory stimulation has been shown to lead to a transient decrease in the vesicular zinc content of neurons that can be visualized with histochemical techniques (Brown and Dyck, 2005). For example, Mitsuya et al. (2009) reported a transient decrease in Timm staining following kainic acid administration to the hippocampus. In our present study, brain tissues were collected on the same day as the seizure latency test. However, in this study, the animals in the four groups were immediately anesthetized for Timm staining and western blot analysis when the mouse first had Racine grade 4 seizures. We did not continue to observe grade 5 seizures. Therefore, there was no difference in the severity of seizures in this experiment. Thus, in this experiment, the effect of the seizure latency test on Timm stainable zinc was the same. The difference in MFS scores in each group still reflects the influence of pilocarpine-induced developmental seizures or PRG5 gene knockout.

Interestingly, treatment of hippocampal HT22 neuron cultures silenced by PRG5, with or without glutamate treatment, markedly elevated intracellular zinc ion levels, and reduced the mitochondrial membrane potential. It has been shown that malfunction or loss of enzymes involved in mitochondrial phospholipid biosynthesis lead to dysfunction of cell respiration, affect the assembly and stability of the mitochondrial protein import machinery and cause abnormal mitochondrial morphology or even lethality (Horvath and Daum, 2013). In addition, mitochondria defect can lead to high-level release of labile Zn2 + from mitochondrial, thereby high intracellular zinc ion level as well as oxidative stress (Turan and Tuncay, 2021). A correlation between epilepsy and cellular redox imbalance has been proposed. Seizure induces mitochondrial oxidative damage and neuronal loss, as well as affect neuronal water and ion balance, thus creating a vicious circle, thereby promoting neuronal hyperexcitability (Pecorelli et al., 2015). Hence, our finding, that PRG5 silencing leads to increased intracellular zinc ion content, and aggravates the damage of mitochondrial function, support the hypothesis that PRG5 knockout precipitates late-onset hypersusceptibility to pilocarpine-induced juvenile seizures by exacerbating hippocampal zinc signaling-mediated mitochondrial damage.

This study has some limitations. It is best to use isolated rat hippocampal neuron cultures combined with a fluorescent Zn2 + sensor and western blot analysis (Sanford et al., 2019) to study the effects of long-term Pgr5 knockdown on hippocampal imaging zinc and the expression changes of zinc transporters, so as to better understand the relationship between Pgr5 and zinc signaling Interaction.

Taken together, PRG5 KO may increase seizure susceptibility. This effect may be related to the hippocampal zinc signaling. The effects do not appear to be related to changes in MFS because KO mice with juvenile seizures had the shortest seizure latencies but exhibited less mossy fiber sprouting than WT mice with juvenile seizures. Further investigations are needed to address this issue.

## Data Availability Statement

The original contributions presented in the study are included in the article/Supplementary Material, further inquiries can be directed to the corresponding author/s.

## Ethics Statement

The animal study was reviewed and approved by the Institutional Animal Care and Use Committee of Soochow University.

## Author Contributions

HN designed the study and wrote the manuscript. DW analyzed the data. DW, YL, LL, M-fJ, and YS were the operators of the experiment and responsible for the statistical analysis of the data. All authors contributed to the article and approved the submitted version.

## Conflict of Interest

The authors declare that the research was conducted in the absence of any commercial or financial relationships that could be construed as a potential conflict of interest.

## Publisher’s Note

All claims expressed in this article are solely those of the authors and do not necessarily represent those of their affiliated organizations, or those of the publisher, the editors and the reviewers. Any product that may be evaluated in this article, or claim that may be made by its manufacturer, is not guaranteed or endorsed by the publisher.
