# Supplementary figures and images for: PRG5 Knockout Precipitates Late-Onset Hypersusceptibility to Pilocarpine-Induced Juvenile Seizures by Exacerbating Hippocampal Zinc Signaling-Mediated Mitochondrial Damage
Source: Front Neurosci. 2021 Aug 27;15:715555. doi: 10.3389/fnins.2021.715555 (PMC8430038; doi:10.3389/fnins.2021.715555)

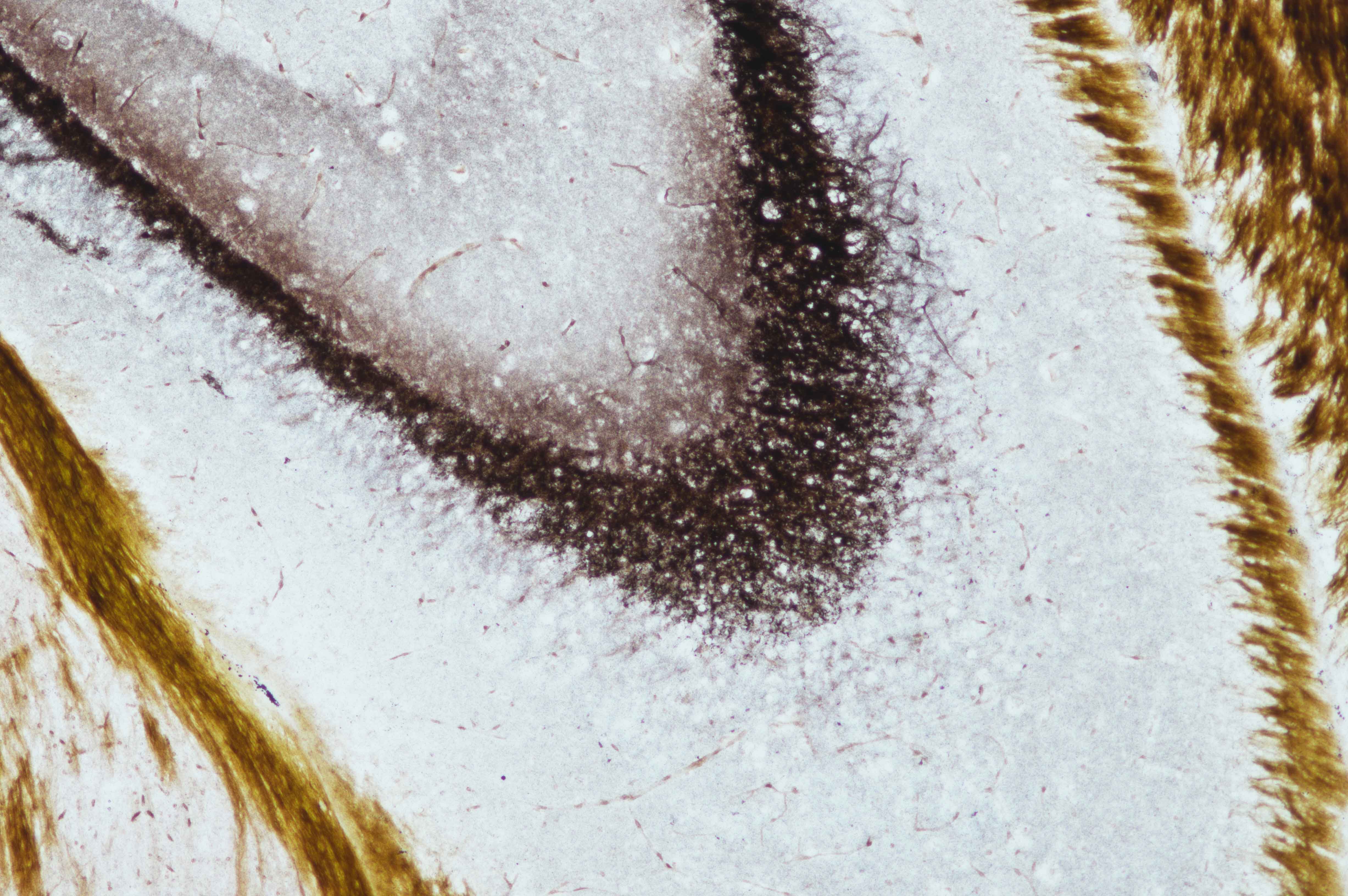

Supplement: Supplementary file 2 [file Data_Sheet_1.ZIP › KO(100).jpg]

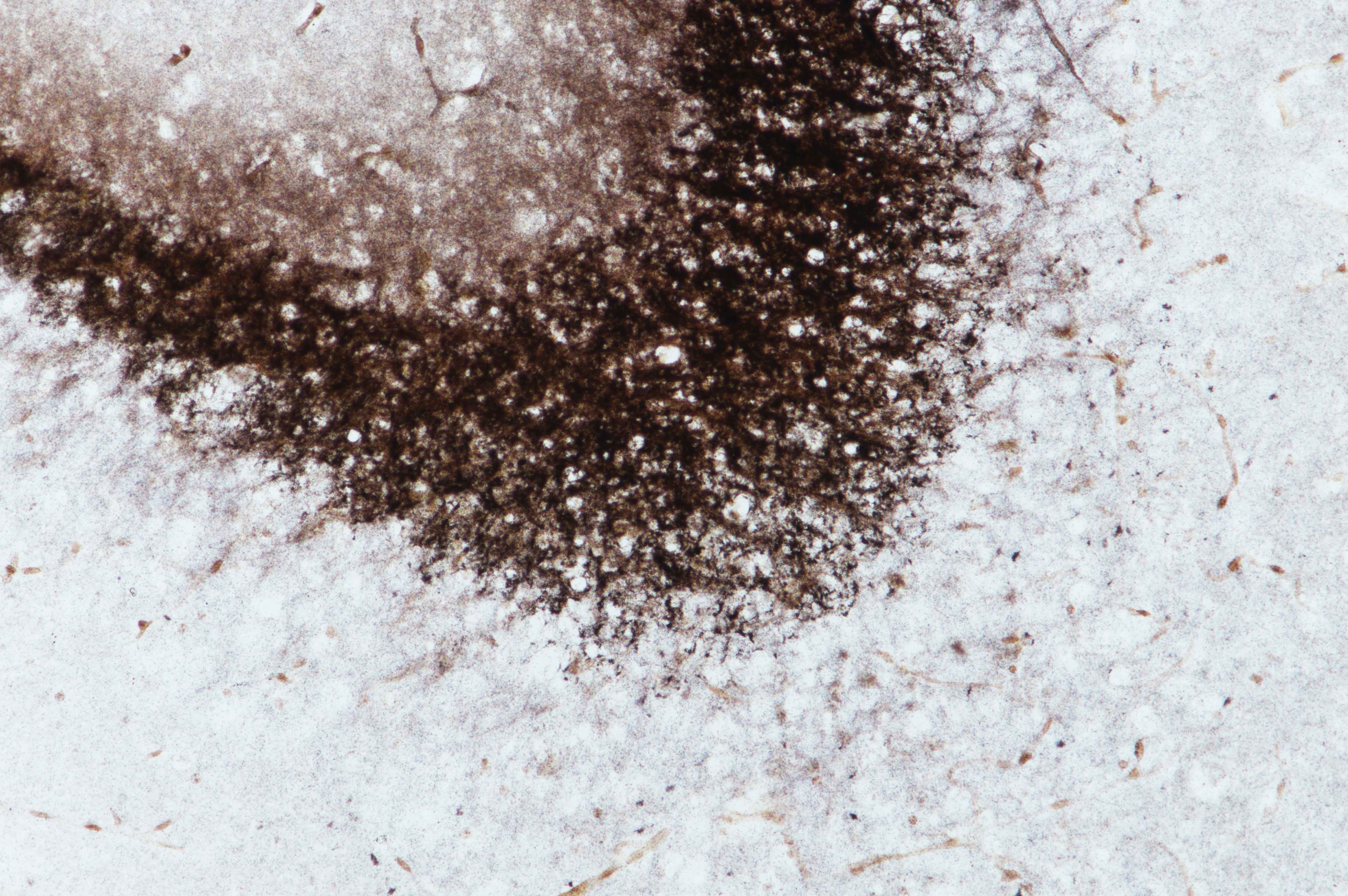

Supplement: Supplementary file 2 [file Data_Sheet_1.ZIP › KO(200).jpg]

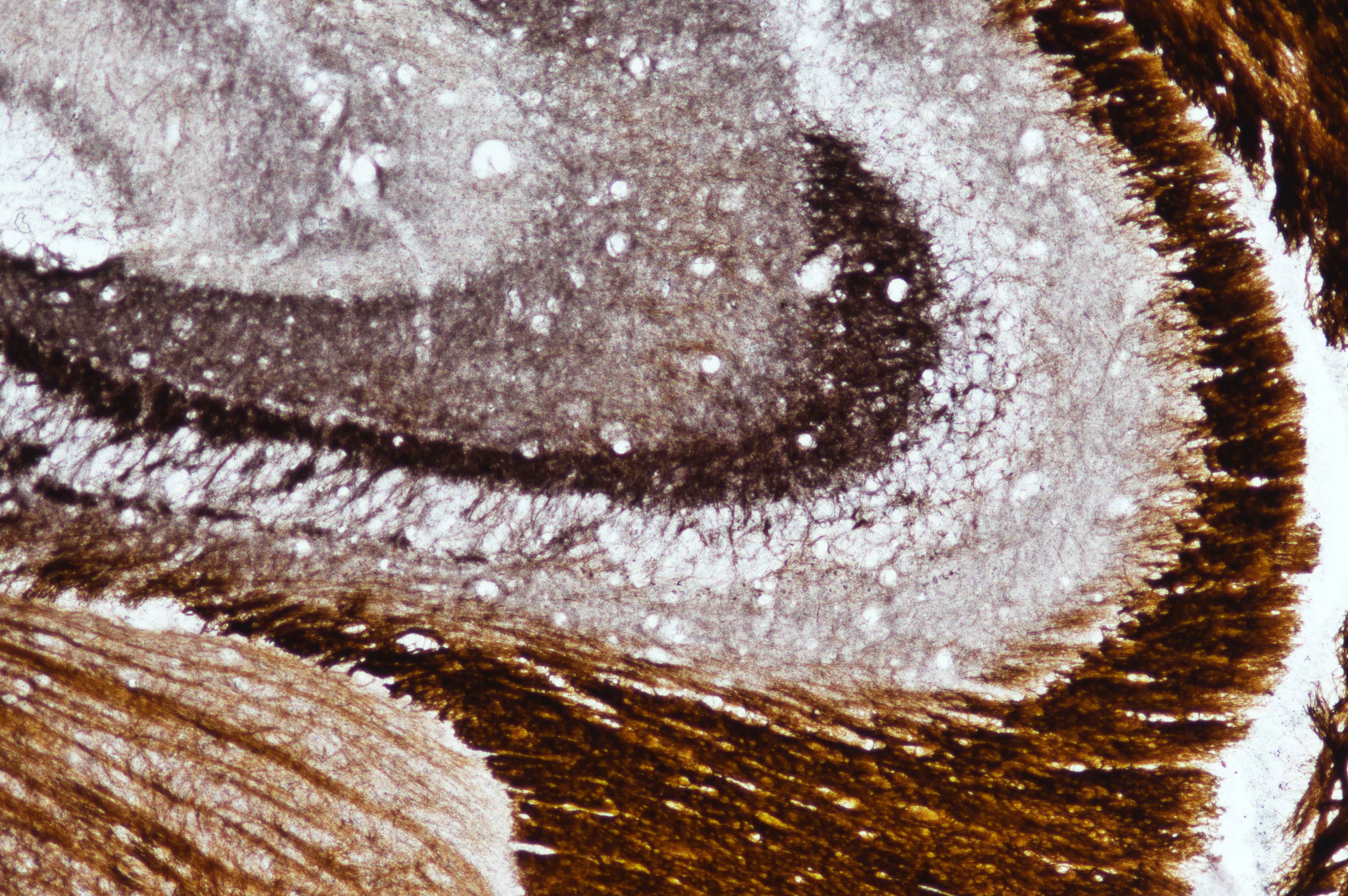

Supplement: Supplementary file 2 [file Data_Sheet_1.ZIP › KO+SE(100).jpg]

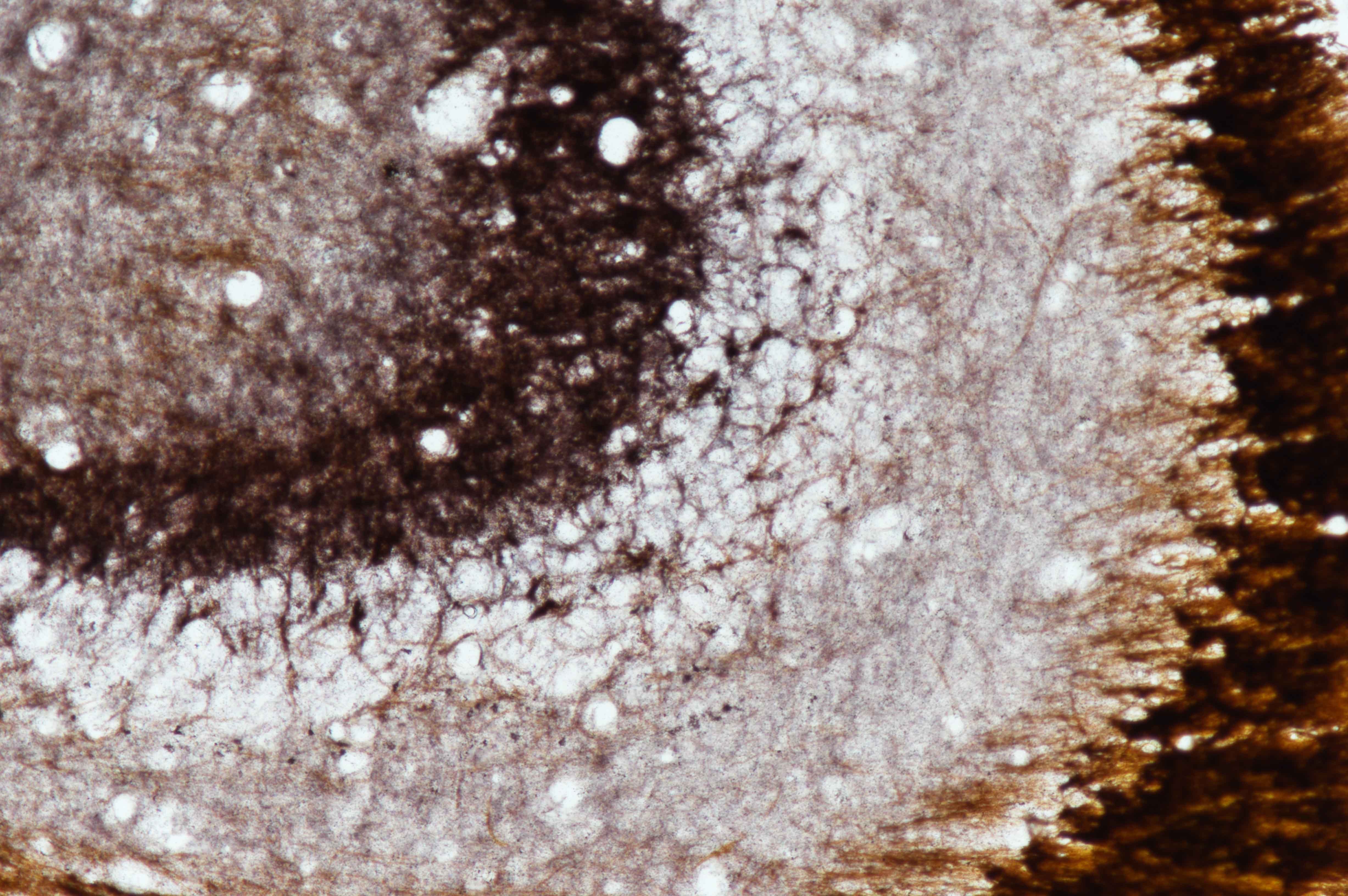

Supplement: Supplementary file 2 [file Data_Sheet_1.ZIP › KO+SE(200).jpg]

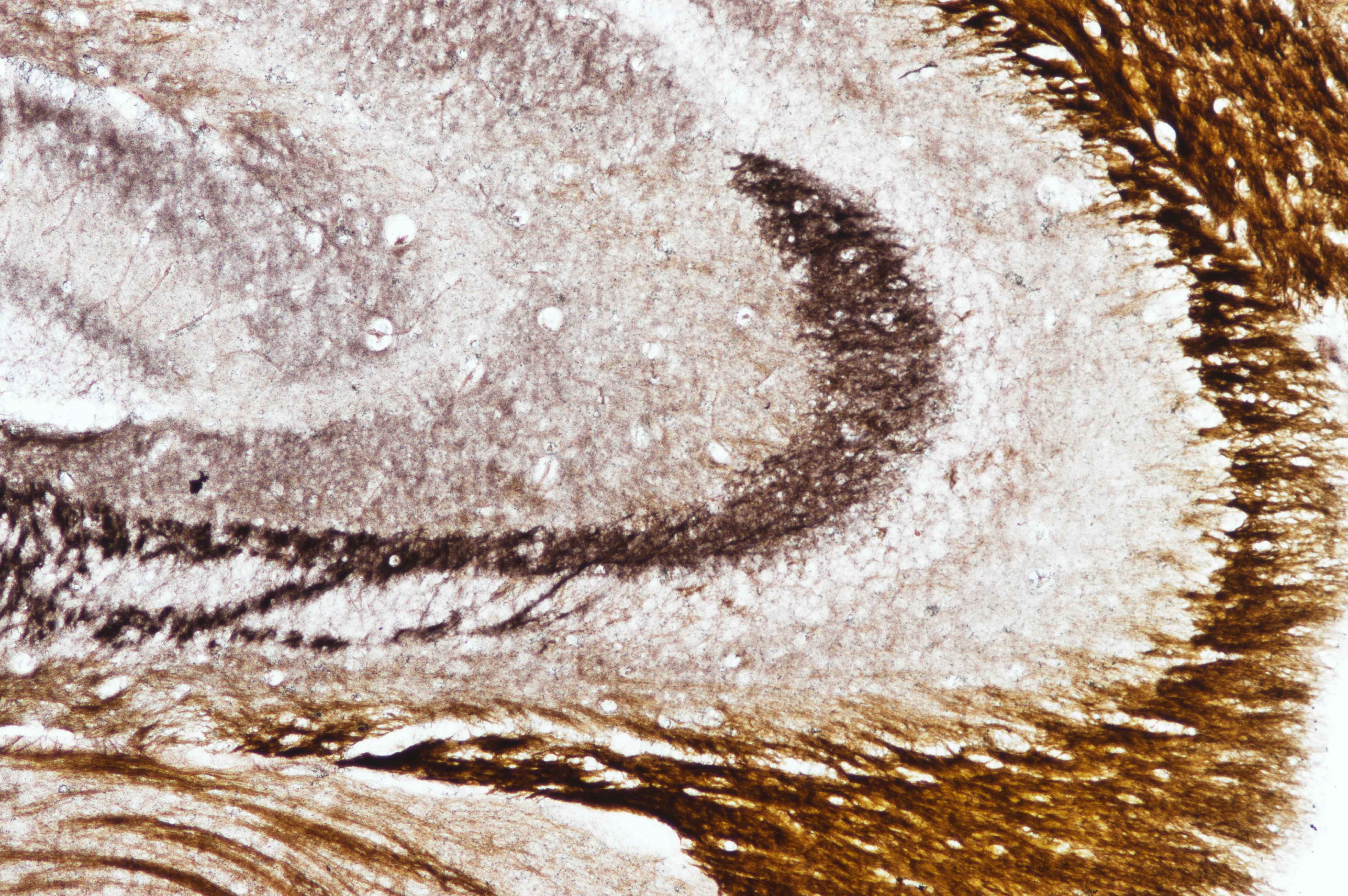

Supplement: Supplementary file 2 [file Data_Sheet_1.ZIP › WT(100).jpg]

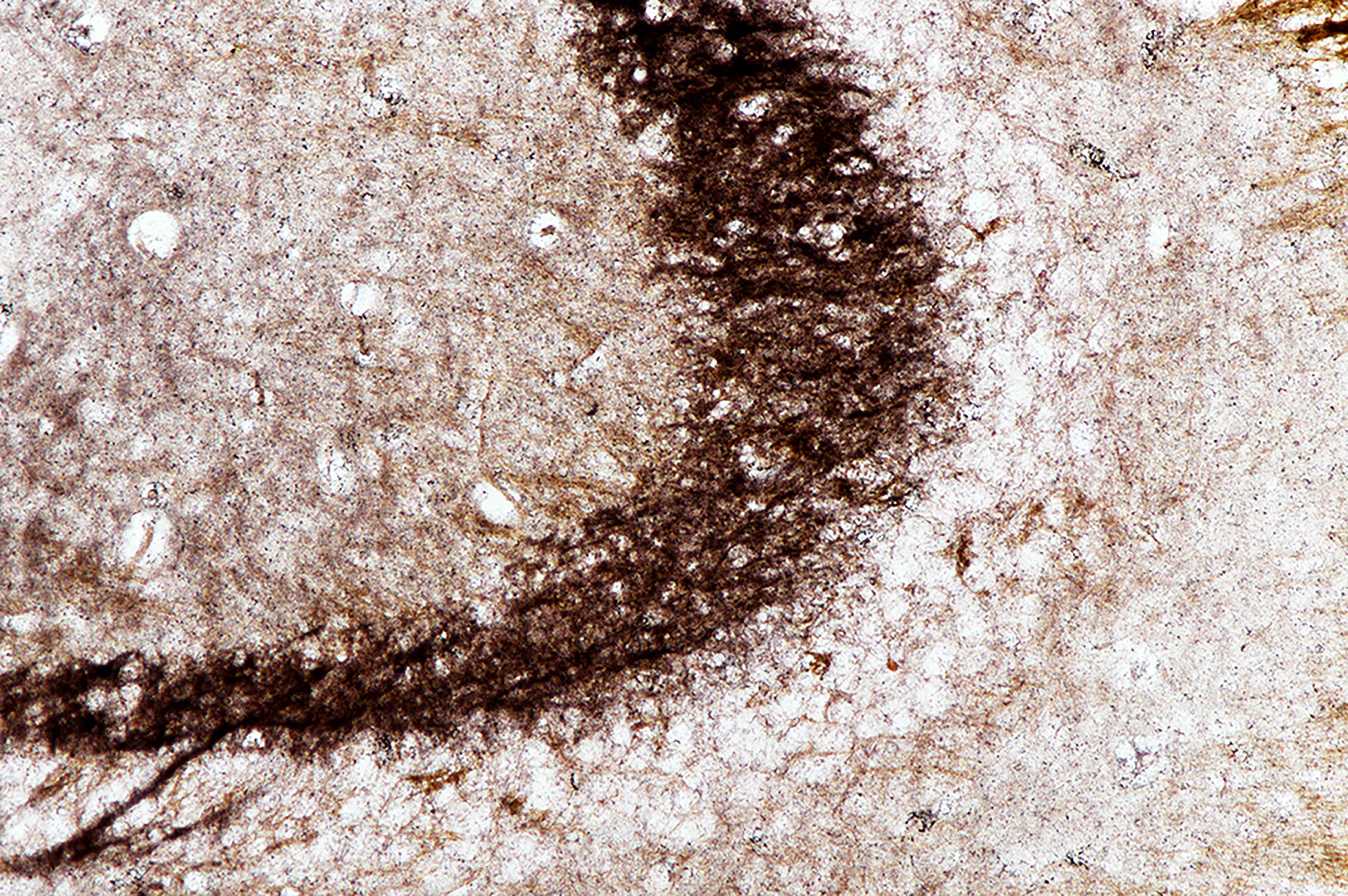

Supplement: Supplementary file 2 [file Data_Sheet_1.ZIP › WT(200).jpg]

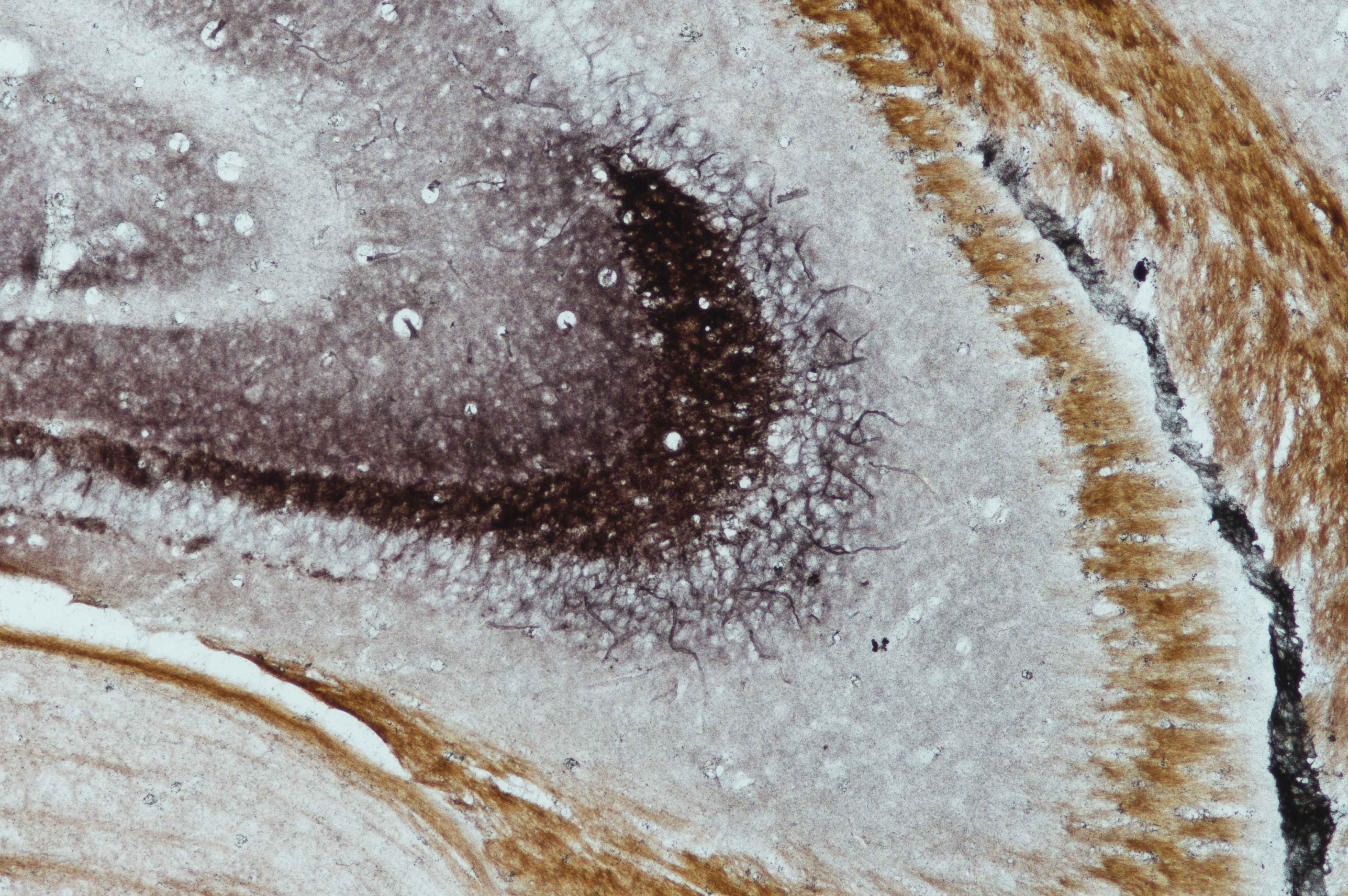

Supplement: Supplementary file 2 [file Data_Sheet_1.ZIP › WT+SE(100).jpg]

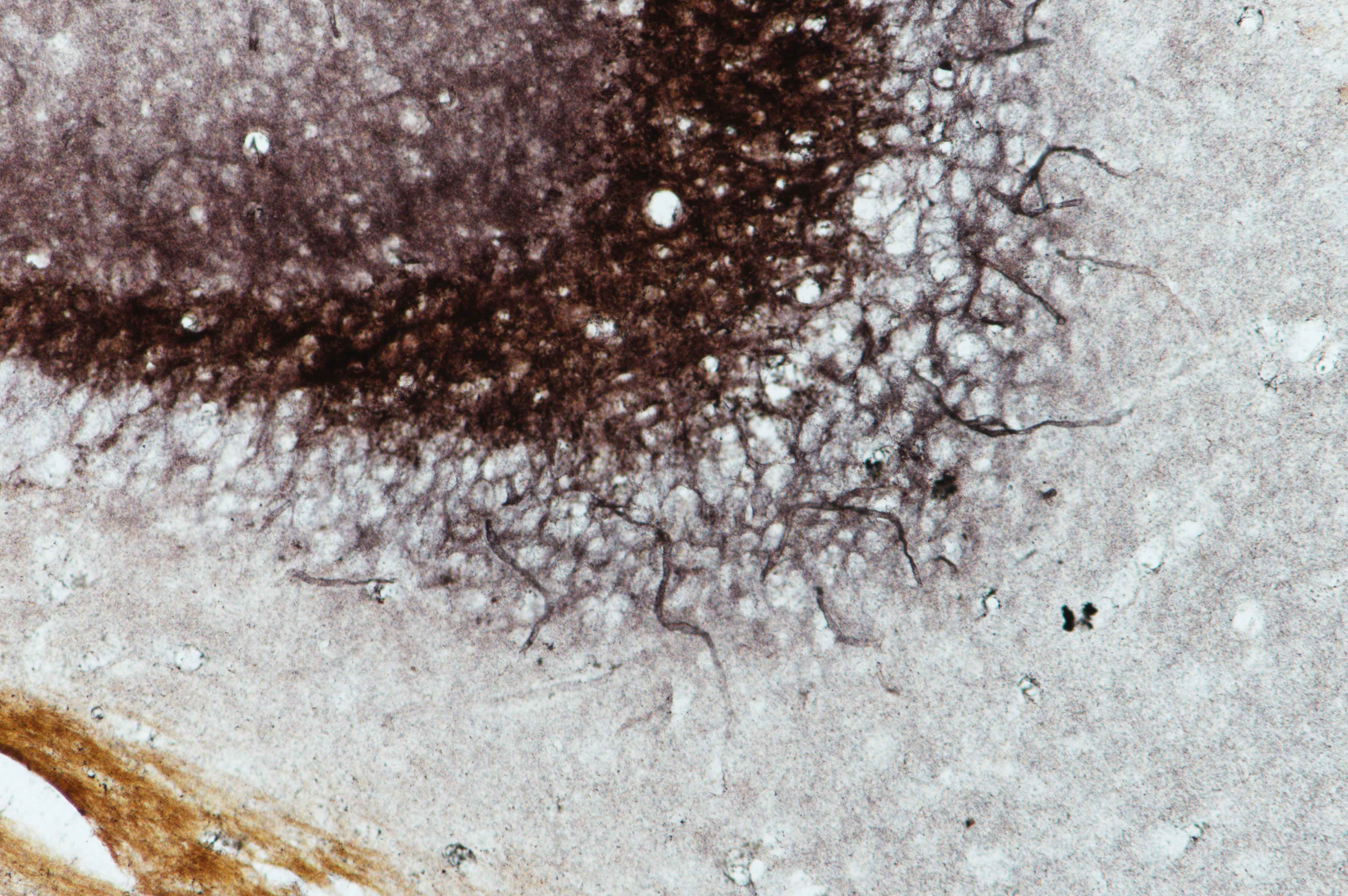

Supplement: Supplementary file 2 [file Data_Sheet_1.ZIP › WT+SE(200).jpg]

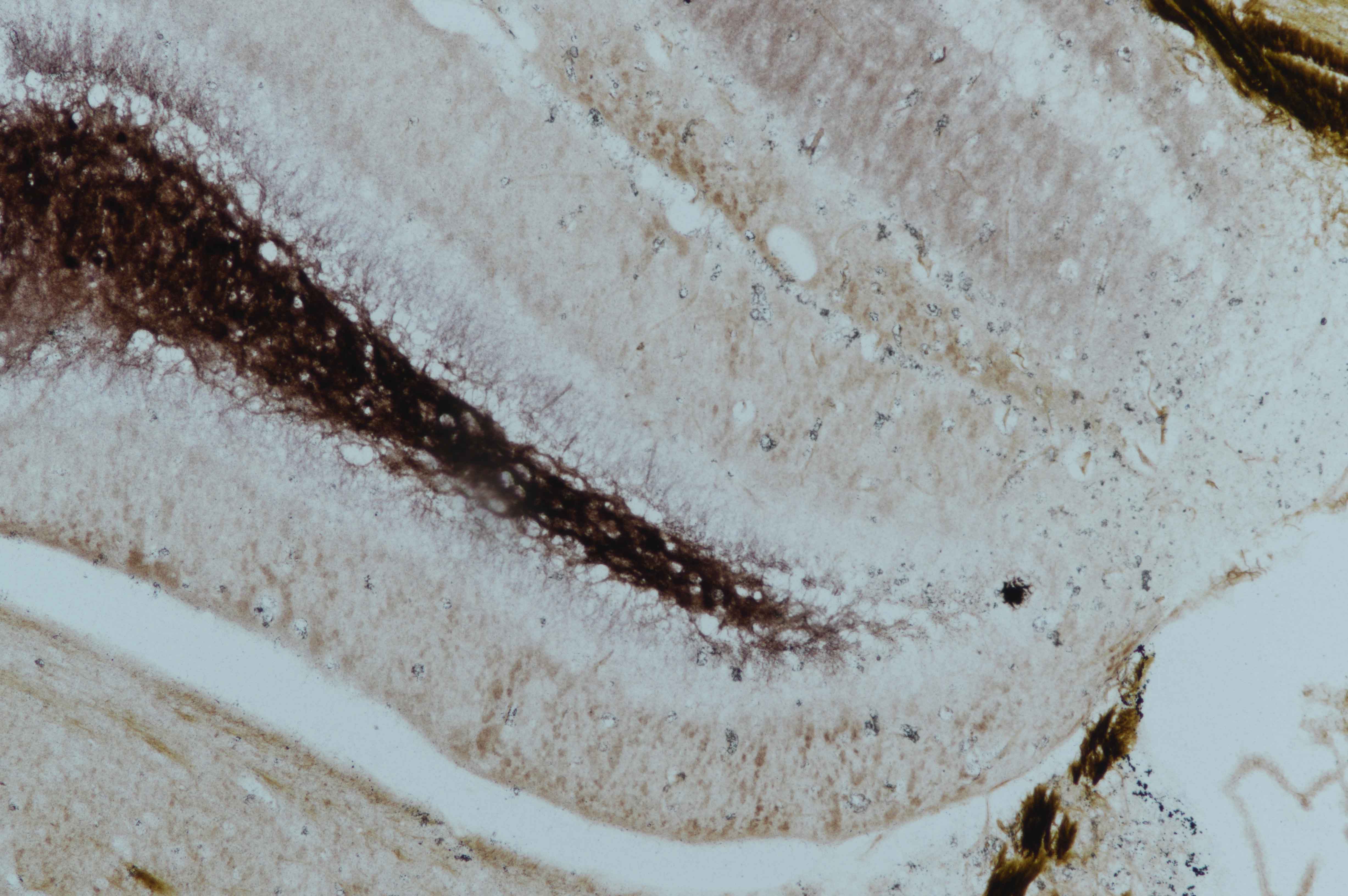

Supplement: Supplementary file 3 [file Data_Sheet_2.ZIP › KO(100).jpg]

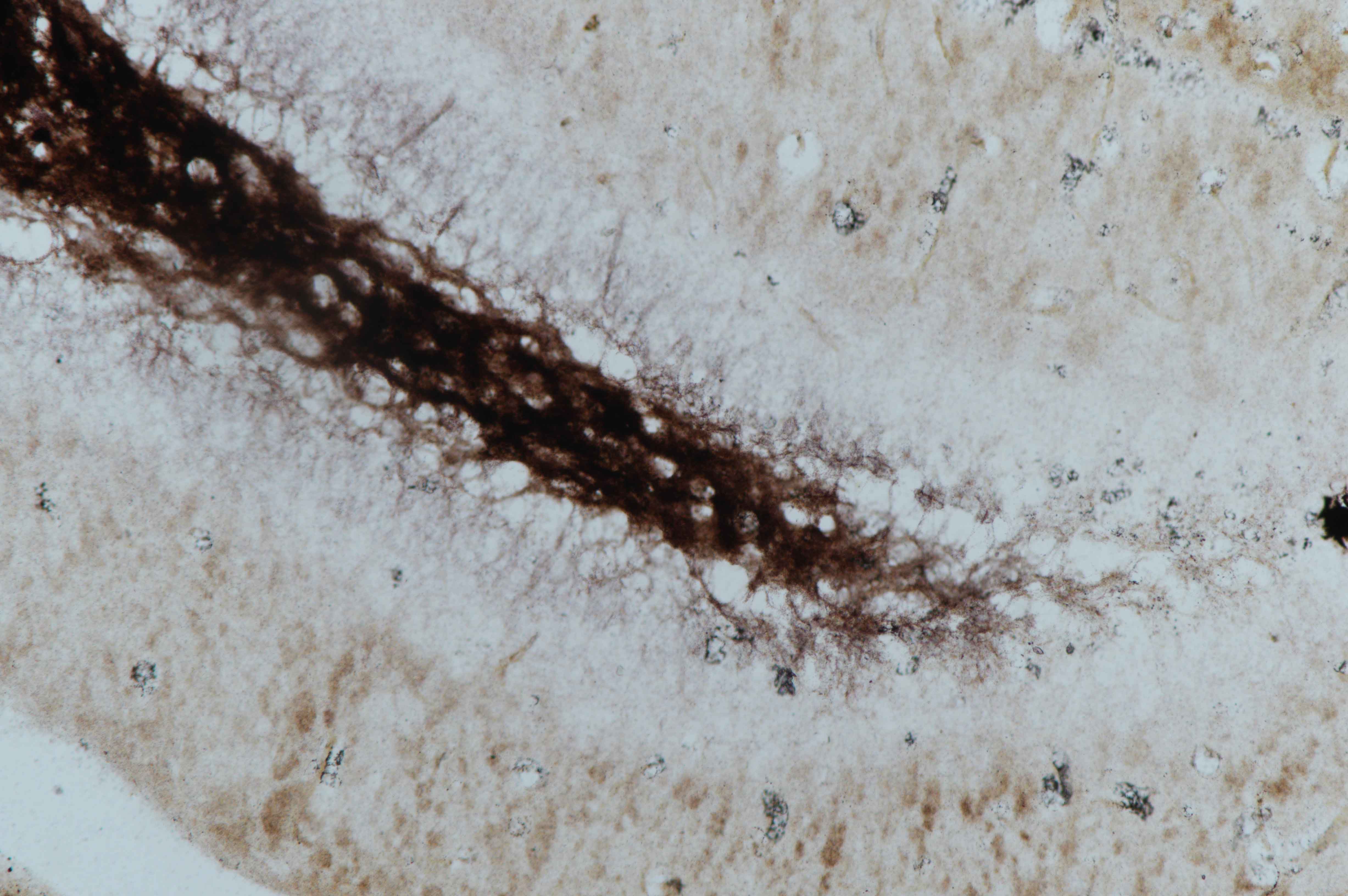

Supplement: Supplementary file 3 [file Data_Sheet_2.ZIP › KO(200).jpg]

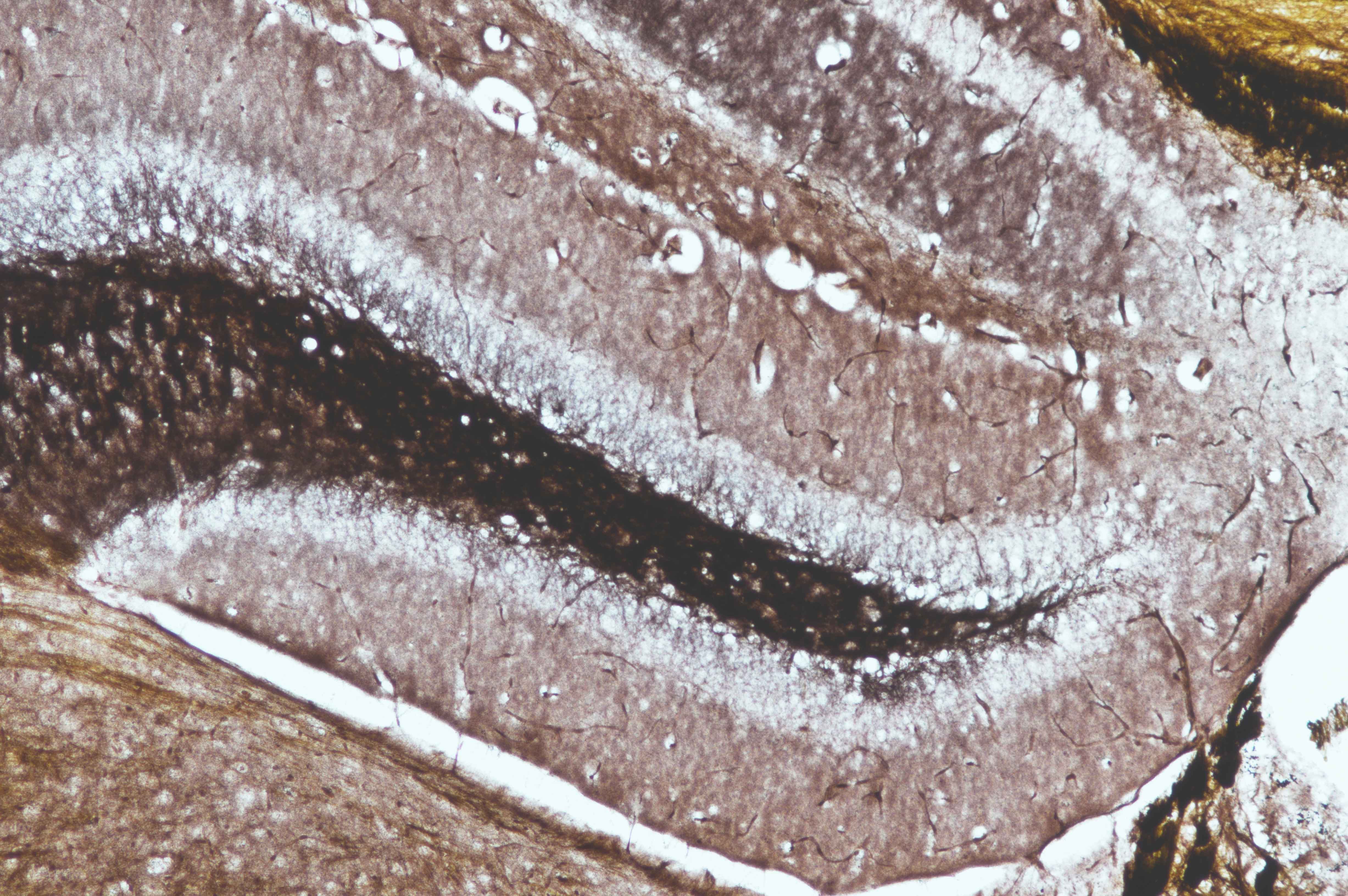

Supplement: Supplementary file 3 [file Data_Sheet_2.ZIP › KO+SE(100).jpg]

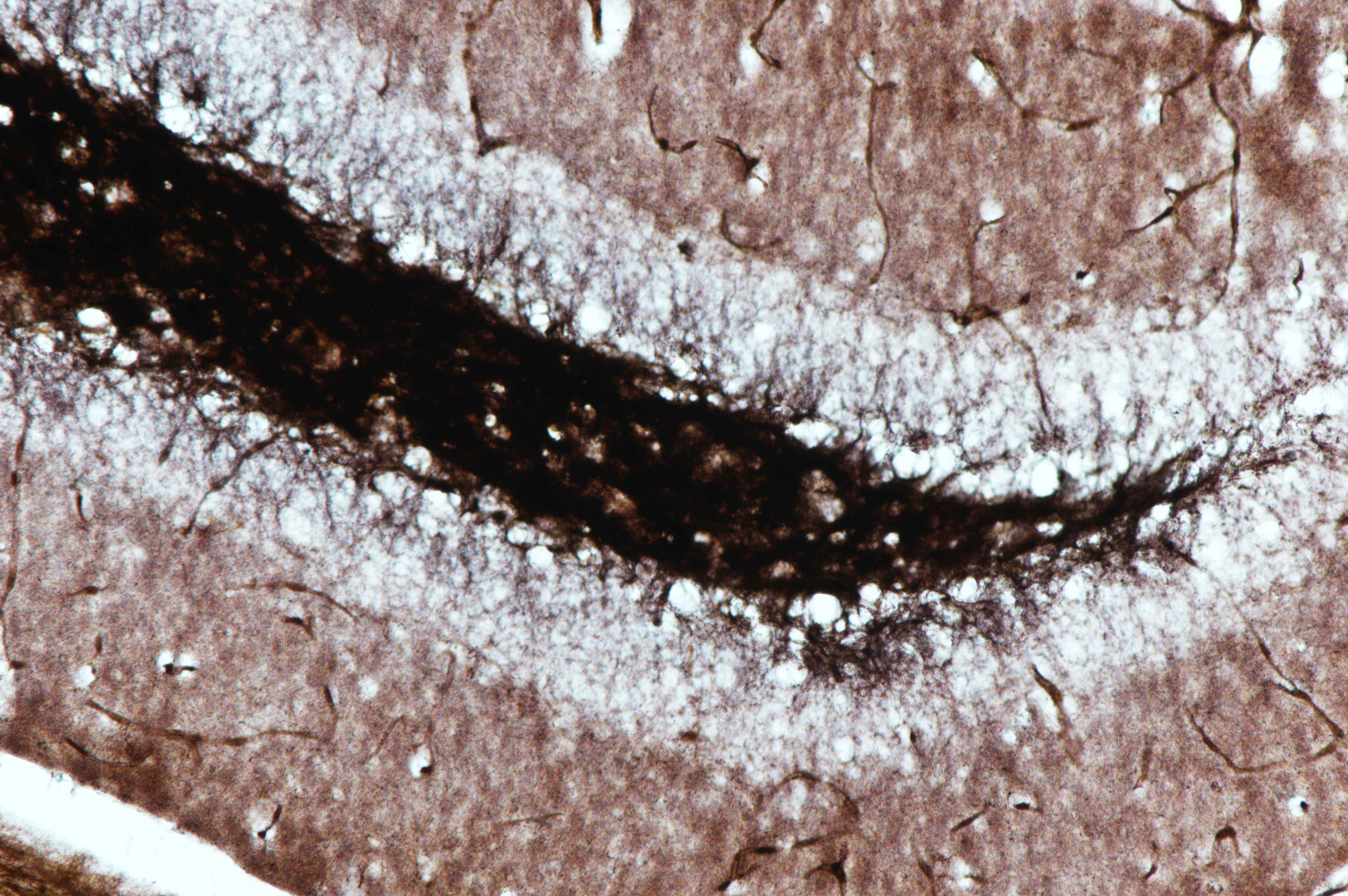

Supplement: Supplementary file 3 [file Data_Sheet_2.ZIP › KO+SE(200).jpg]

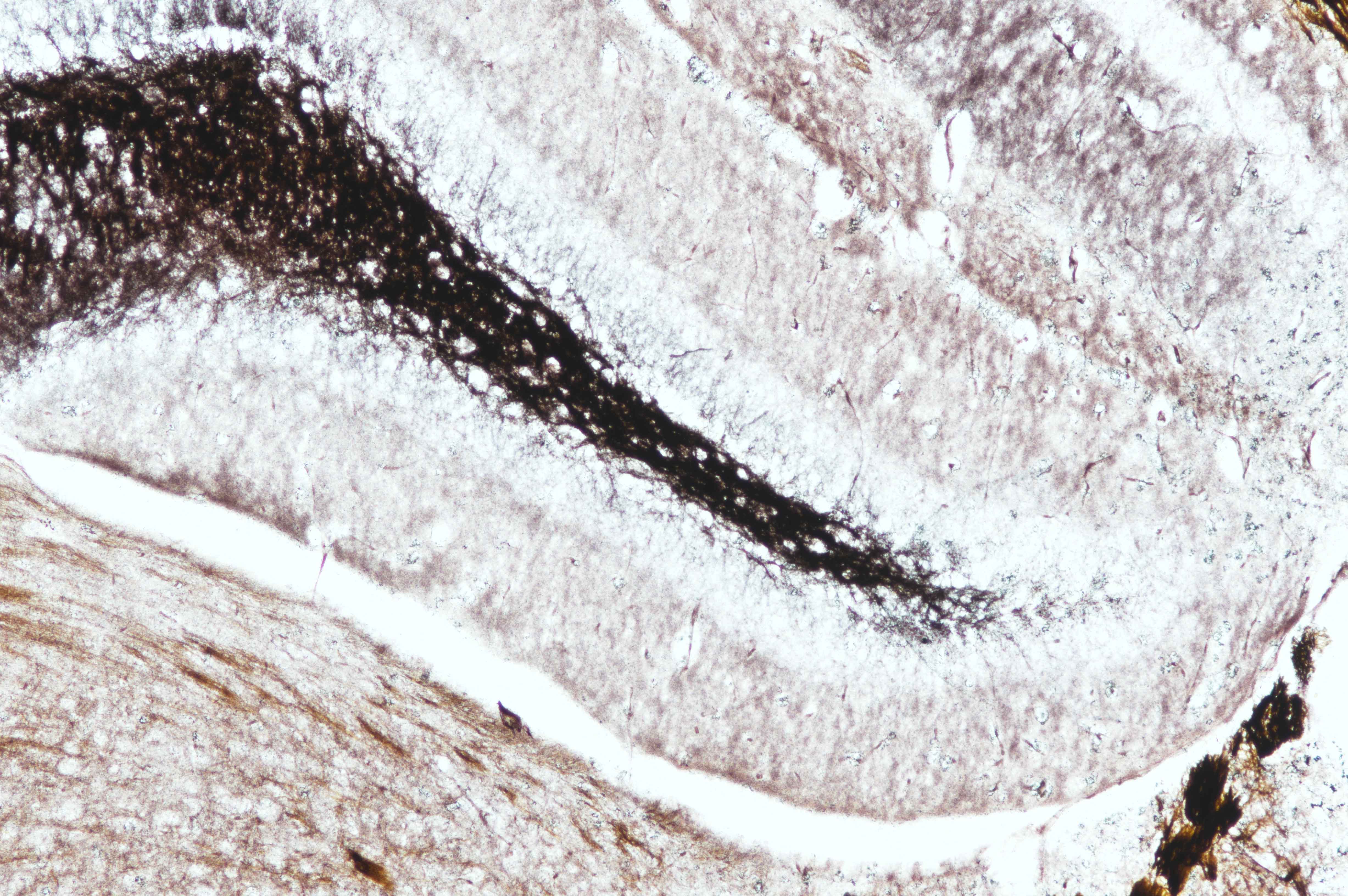

Supplement: Supplementary file 3 [file Data_Sheet_2.ZIP › WT(100).jpg]

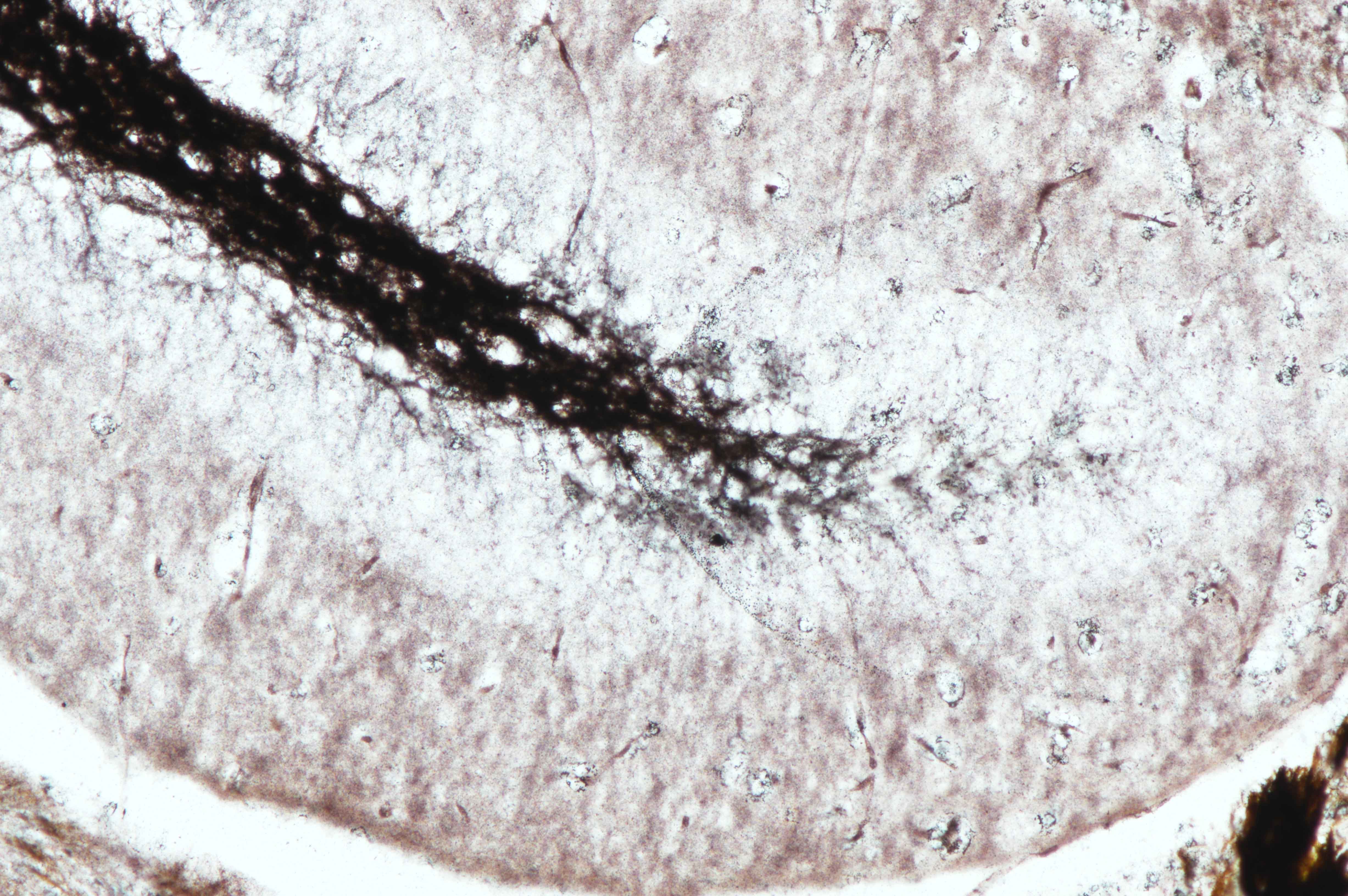

Supplement: Supplementary file 3 [file Data_Sheet_2.ZIP › WT(200).jpg]

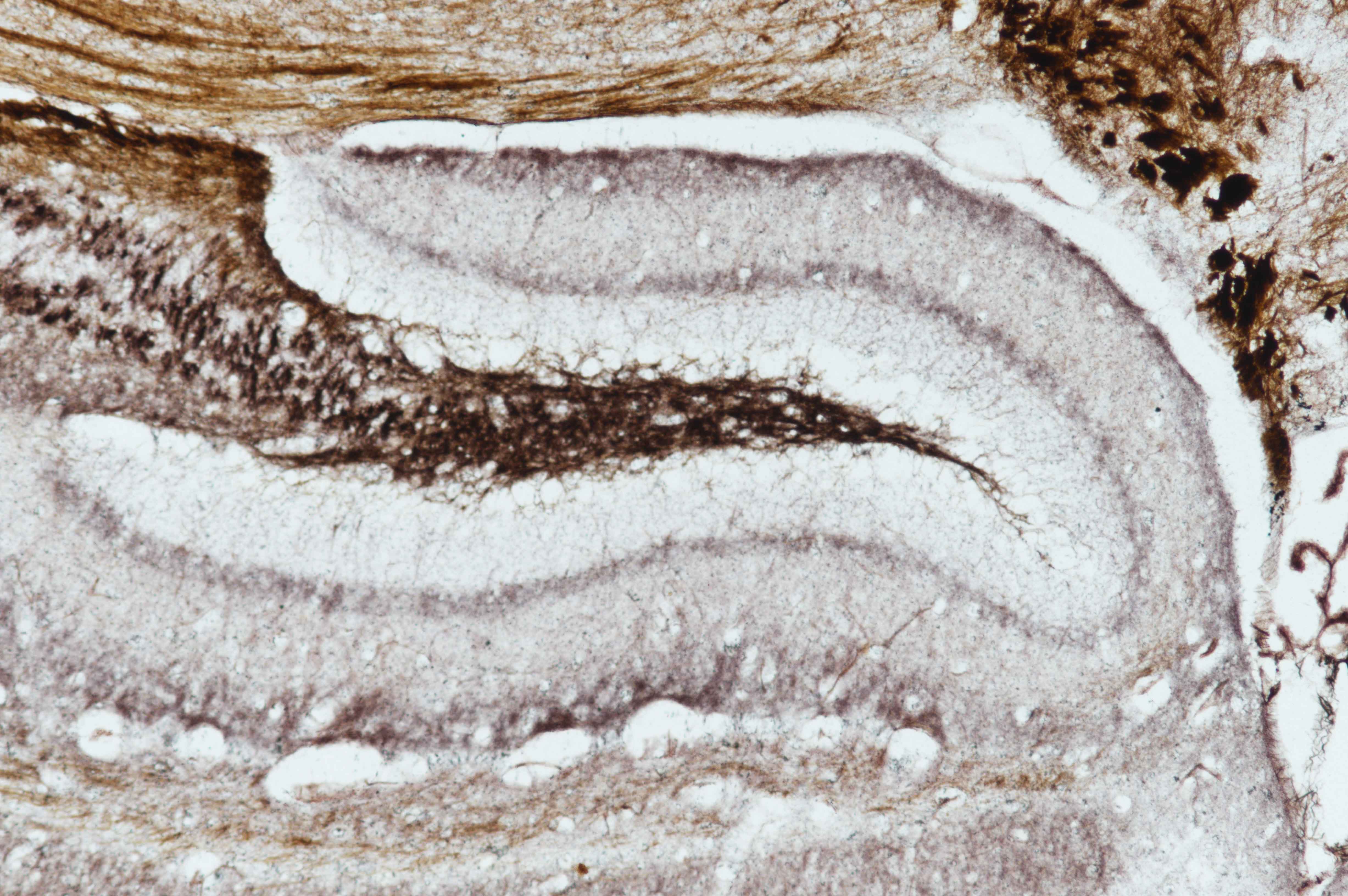

Supplement: Supplementary file 3 [file Data_Sheet_2.ZIP › WT+SE(100).jpg]

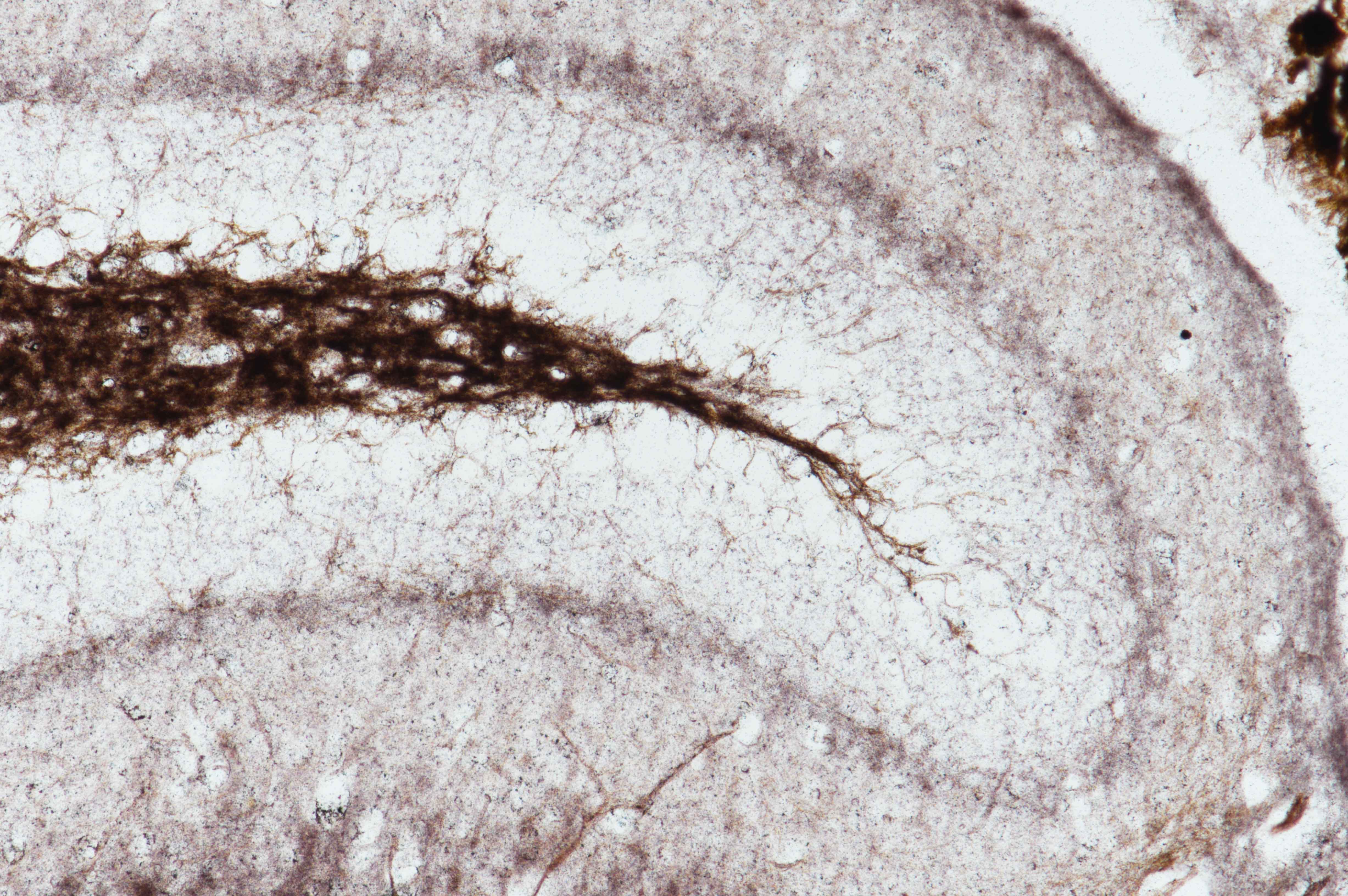

Supplement: Supplementary file 3 [file Data_Sheet_2.ZIP › WT+SE(200).jpg]

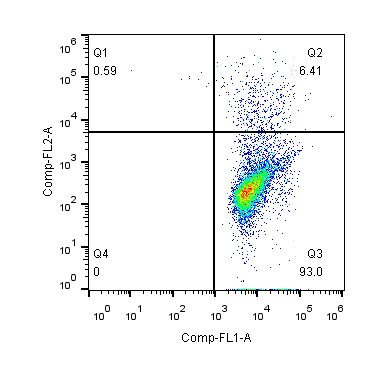

Supplement: Supplementary file 4 [file Data_Sheet_3.ZIP › shPRG5+Glutamate.jpg]

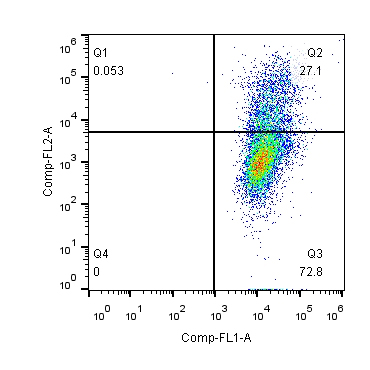

Supplement: Supplementary file 4 [file Data_Sheet_3.ZIP › shControl.jpg]

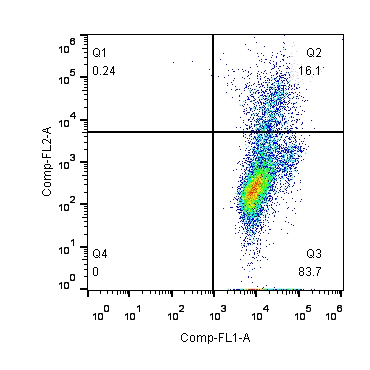

Supplement: Supplementary file 4 [file Data_Sheet_3.ZIP › shControl+Glutamate.jpg]

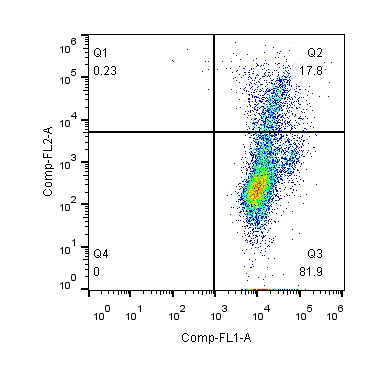

Supplement: Supplementary file 4 [file Data_Sheet_3.ZIP › shPRG5.jpg]

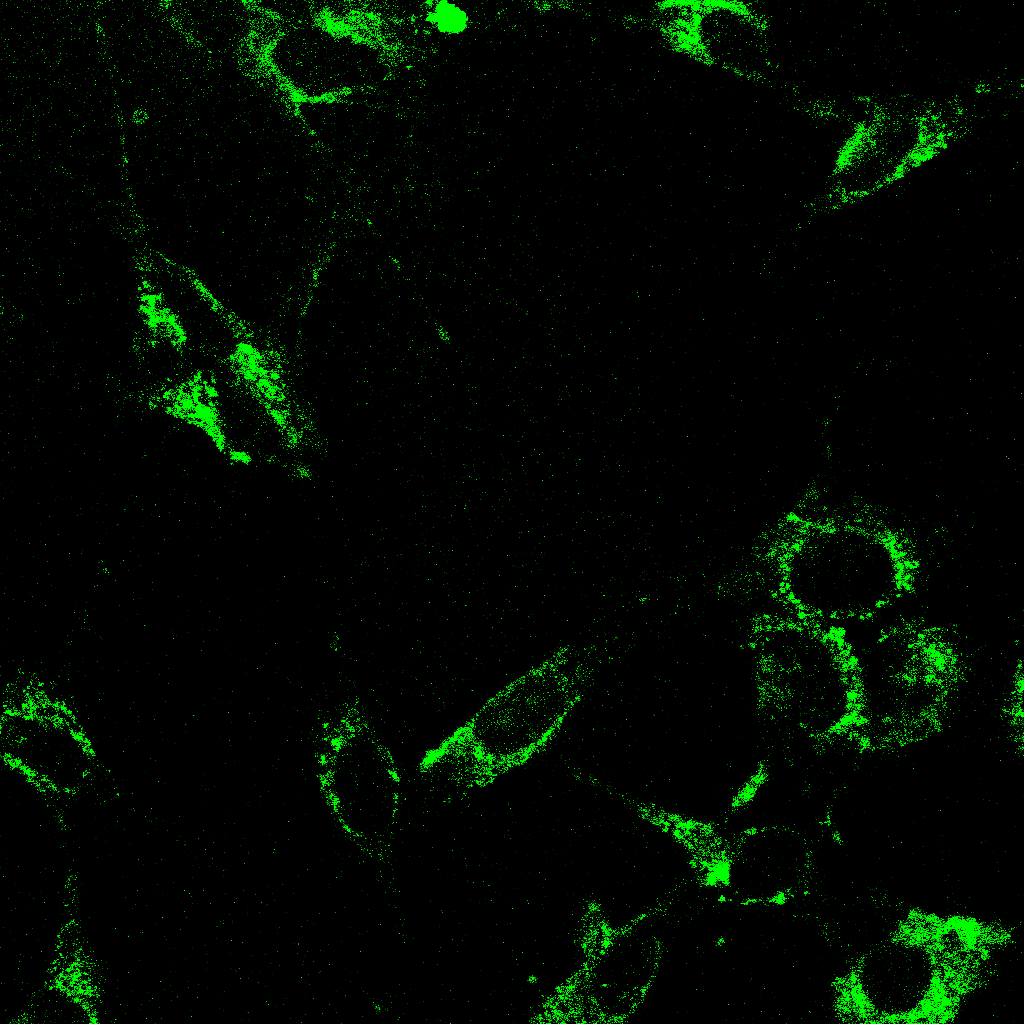

Supplement: Supplementary file 5 [file Data_Sheet_4.ZIP › shPRG5+Glutamate.tif]

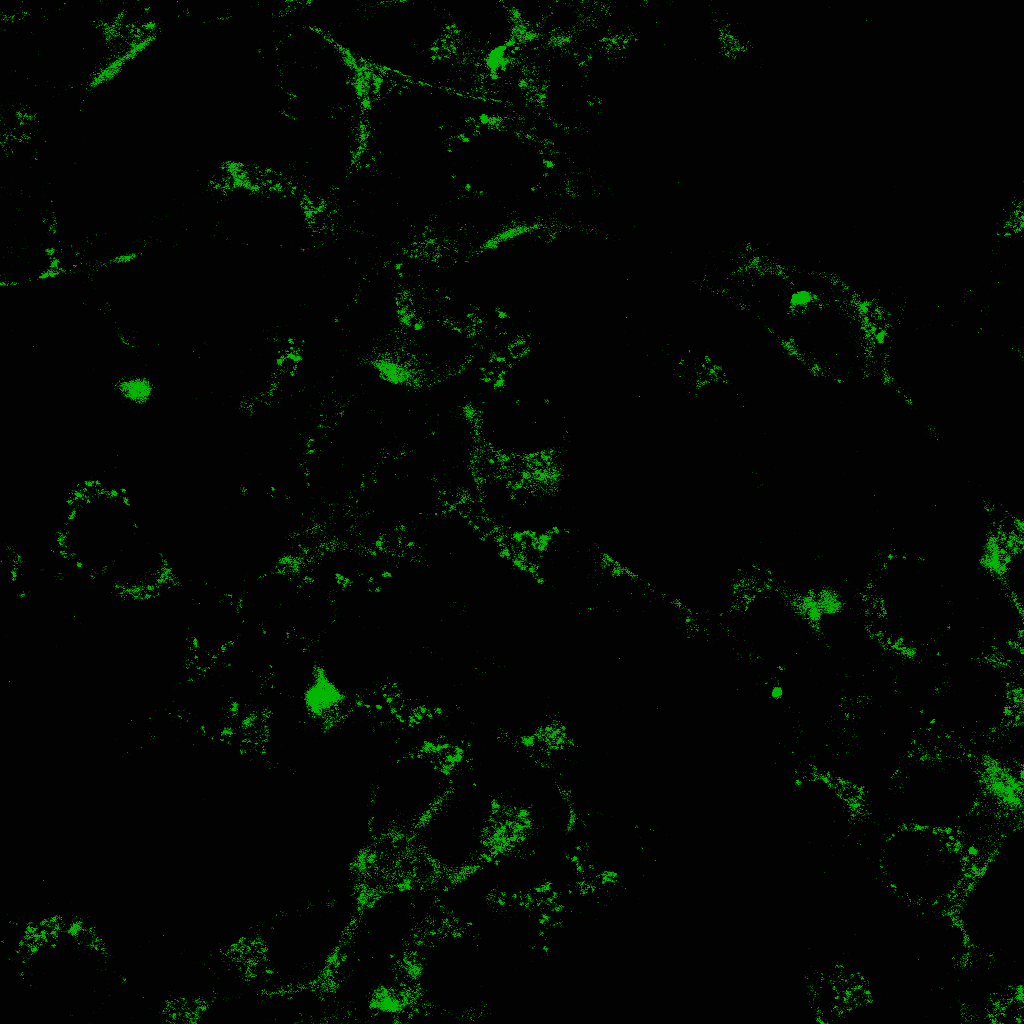

Supplement: Supplementary file 5 [file Data_Sheet_4.ZIP › shControl+Glutamate.tif]

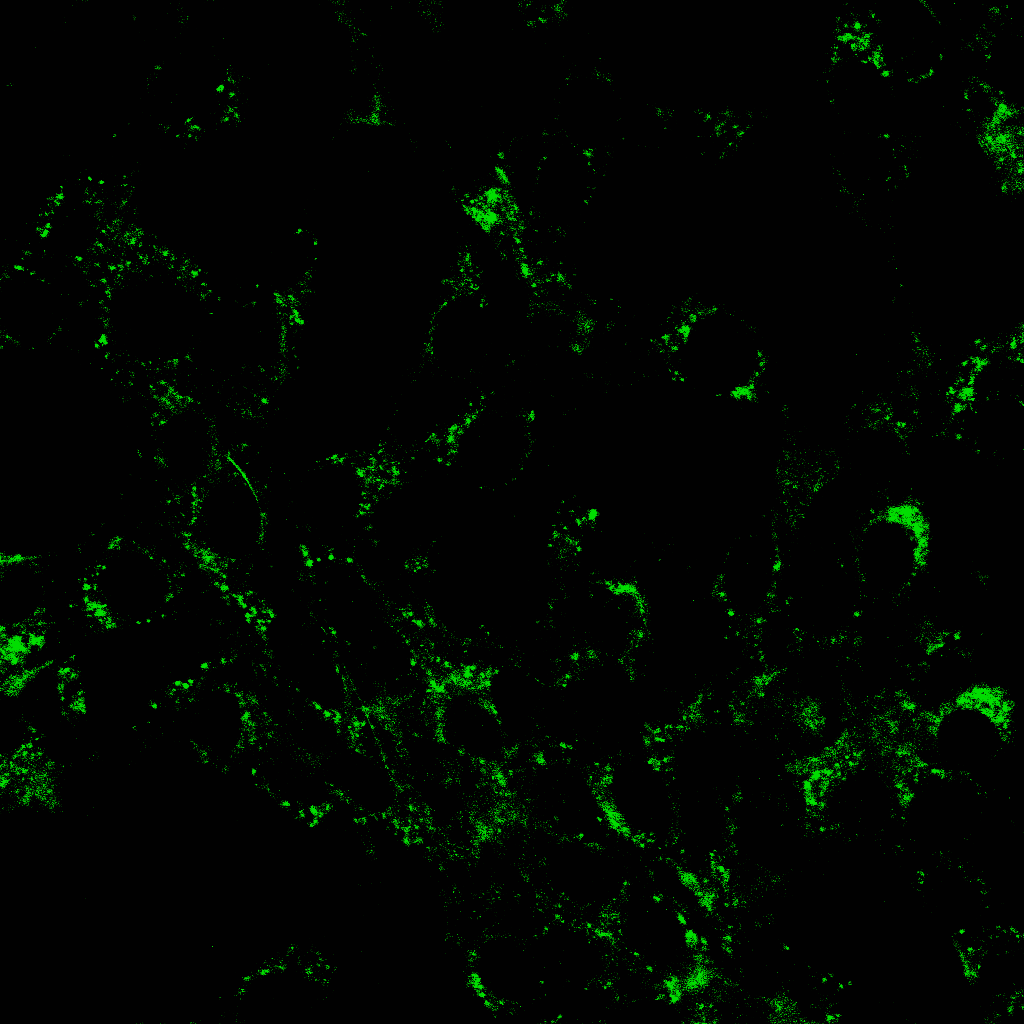

Supplement: Supplementary file 5 [file Data_Sheet_4.ZIP › shPRG5.tif]

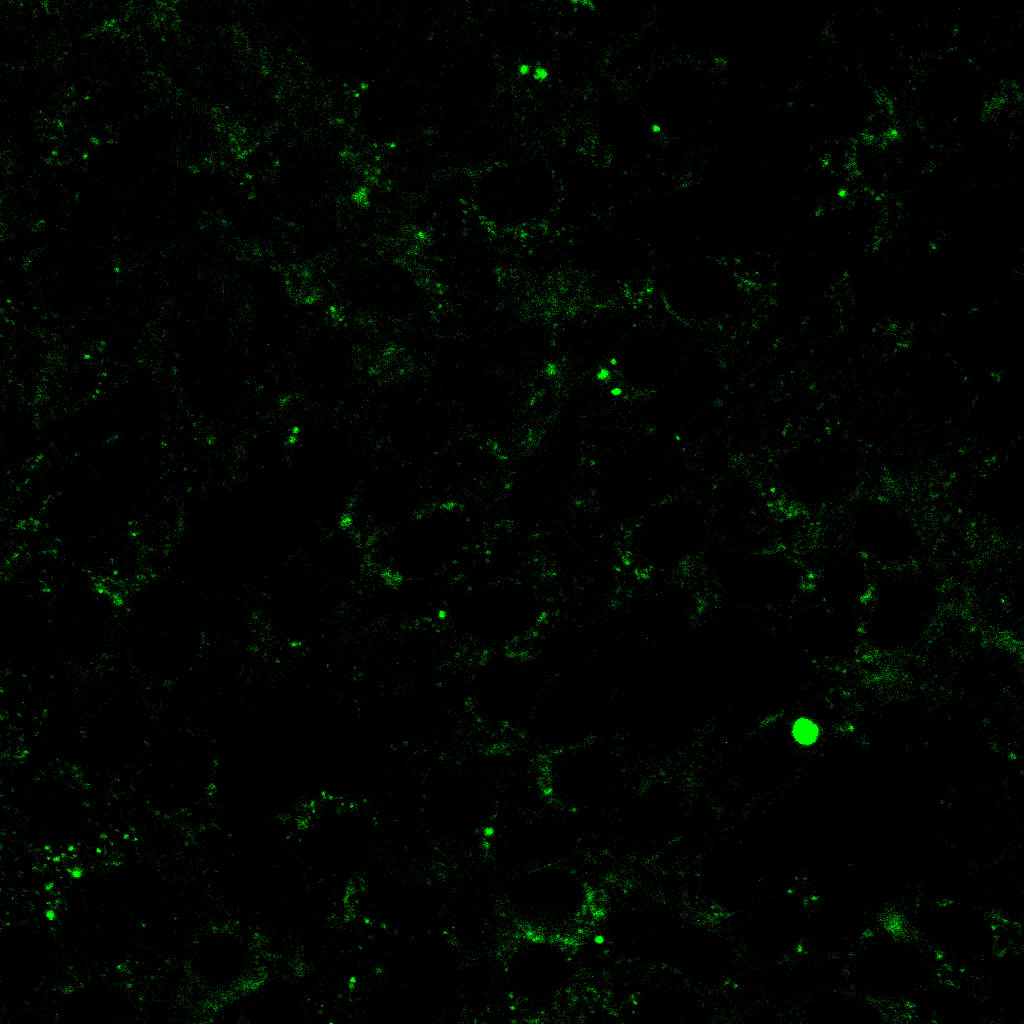

Supplement: Supplementary file 5 [file Data_Sheet_4.ZIP › shControl.tif]
